# Genome-scale Co-evolutionary Inference Identifies Functions and Clients of Bacterial Hsp90

**DOI:** 10.1371/journal.pgen.1003631

**Published:** 2013-07-11

**Authors:** Maximilian O. Press, Hui Li, Nicole Creanza, Günter Kramer, Christine Queitsch, Victor Sourjik, Elhanan Borenstein

**Affiliations:** 1 Department of Genome Sciences, University of Washington, Seattle, Washington, United States of America; 2 Zentrum für Molekulare Biologie der Universität Heidelberg, DKFZ-ZMBH Alliance, Heidelberg, Germany; 3 Department of Biology, Stanford University, Stanford, California, United States of America; 4 Department of Computer Science and Engineering, University of Washington, Seattle, Washington, United States of America; 5 Santa Fe Institute, Santa Fe, New Mexico, United States of America; Université Paris Descartes, INSERM U1001, France

## Abstract

The molecular chaperone Hsp90 is essential in eukaryotes, in which it facilitates the folding of developmental regulators and signal transduction proteins known as Hsp90 clients. In contrast, Hsp90 is not essential in bacteria, and a broad characterization of its molecular and organismal function is lacking. To enable such characterization, we used a genome-scale phylogenetic analysis to identify genes that co-evolve with bacterial Hsp90. We find that genes whose gain and loss were coordinated with Hsp90 throughout bacterial evolution tended to function in flagellar assembly, chemotaxis, and bacterial secretion, suggesting that Hsp90 may aid assembly of protein complexes. To add to the limited set of known bacterial Hsp90 clients, we further developed a statistical method to predict putative clients. We validated our predictions by demonstrating that the flagellar protein FliN and the chemotaxis kinase CheA behaved as Hsp90 clients in *Escherichia coli*, confirming the predicted role of Hsp90 in chemotaxis and flagellar assembly. Furthermore, normal Hsp90 function is important for wild-type motility and/or chemotaxis in *E. coli*. This novel function of bacterial Hsp90 agreed with our subsequent finding that Hsp90 is associated with a preference for multiple habitats and may therefore face a complex selection regime. Taken together, our results reveal previously unknown functions of bacterial Hsp90 and open avenues for future experimental exploration by implicating Hsp90 in the assembly of membrane protein complexes and adaptation to novel environments.

## Author Summary

Hsp90 is a chaperone protein that aids the folding of many other proteins (clients), which tend to be signal transduction proteins. Hsp90 is particularly important when organisms are under environmental or mutational stress (e.g. in cancerous cells). Although Hsp90 is well-studied in eukaryotic species from yeast to humans, little is known about its counterpart in bacteria. To address this challenge, we analyzed the presence and absence of thousands of genes across numerous bacterial species and identified genes that co-evolved with Hsp90. These genes provide insights into potential functions of Hsp90 in bacteria. We found that Hsp90 co-evolves with membrane-associated protein complexes such as the flagellum and that Hsp90 is associated with a preference for inhabiting multiple habitats. We extended our analysis to identify genes that exhibit evolutionary dynamics characteristic of Hsp90 clients. Many of the putative clients were involved in flagellar assembly, suggesting a crucial role of Hsp90 in the regulation of bacterial motility. We experimentally confirmed that *E. coli* Hsp90 interacts with selected candidates and demonstrated Hsp90's role in flagellar motility and chemotaxis. The computational approach described here, identifying novel functions and specific clients of bacterial Hsp90, further provides exciting starting points for research in bacterial chaperone biology.

## Introduction

In eukaryotes, the universally conserved and essential chaperone Hsp90 aids the folding of key proteins in development and responses to environmental stimuli [1]–[3]. In yeast, up to 10% of all proteins are estimated to be Hsp90 clients under standard culture conditions [4]. Hsp90 function is even more important under stressful conditions that challenge protein folding, such as increased temperature [5]. The activity of eukaryotic Hsp90 is further modulated by various co-chaperones, which confer substrate specificity and alter protein folding kinetics [2], [5]. Depletion of eukaryotic Hsp90 *in vivo* increases phenotypic variation, reveals ‘cryptic’ heritable variation, and increases penetrance of mutations [6]–[9]. Accordingly, eukaryotic Hsp90 enables organisms to maintain a stable phenotype in the face of environmental and genetic perturbation and to correctly interpret environmental stimuli.

In stark contrast, in prokarya, Hsp90 is not essential [10] and many bacterial genomes lack Hsp90 altogether [11]. Among Archaea, only very few species contain Hsp90, and those are thought to have gained Hsp90 horizontally from bacteria [11], [12]. This fragmented phylogenetic pattern likely results from multiple independent gains and losses, though phylogenetic reconstructions are confused by ancient Hsp90 paralogy [11], [12]. At the amino acid level, the *Escherichia coli* Hsp90 (High-temperature protein G or HtpG) is 42% identical to its human homolog, suggesting strong stabilizing selection consistent with functional conservation [13]. Indeed, *E. coli* Hsp90 appears to retain generic protein chaperone activity [14] and homologous Hsp90 mutations cause chaperone defects in both the prokaryotic *E. coli* and eukaryotic yeast [15]. However, there are no identified obligate Hsp90 co-chaperones in bacteria, adding to the uncertainty regarding the extent of its client spectrum and specificity.

To date, only three proteins have been implicated as Hsp90 clients in bacteria, with non-overlapping functions in ribosome assembly, the assembly of light-harvesting complexes, and the CRISPR/Cas immunity system [16]–[18]. Several other proteins have been shown to physically interact with the chaperone [19], [20]. Together with our knowledge of eukaryotic Hsp90 function, these data have given rise to the speculation that Hsp90 may facilitate the assembly of oligomeric protein complexes in bacteria, much like it does in eukaryotes [21]. Unlike in eukaryotes, however, further exploration of Hsp90's functional role in bacteria has proven challenging because there are no pleiotropic Hsp90-dependent phenotypes.

To address this challenge, we used a genome-scale co-evolutionary ‘guilt-by-association’ approach [22], [23] to explore the spectrum of conserved Hsp90-associated genes, functions, and organismal traits. Hsp90-associated genes tended to function in flagellar assembly, chemotaxis, and secretion. Consistent with these functions, Hsp90-associated organismal traits included the ability to inhabit multiple environments. To add to the sparse list of known bacterial Hsp90 clients, we further developed a statistical method to predict putative Hsp90 clients, which included flagellar, ribosomal, and chaperone proteins. We validated our predictions experimentally, focusing on two candidates functioning in motility and chemotaxis. Indeed, both the flagellar protein FliN and the kinase CheA were found to be Hsp90 clients *in vivo*. Our findings demonstrate the power of co-evolutionary inference to correctly identify substrates and functions of conserved genes like bacterial Hsp90.

## Results

### Hsp90 paralogs in bacteria

Our method for inferring the function of bacterial Hsp90 is based on the analysis of its distribution across the bacterial phylogeny. However, this analysis is complicated by the existence of multiple ancient Hsp90 paralogs in bacteria. These paralogs may be older than existing phyla in bacteria [11], [12], and may have evolved distinct functions on this enormous time scale. To address this issue and to identify each paralog, we first clustered bacterial Hsp90s by sequence identity. We identified 897 bacterial Hsp90 protein sequences in the KEGG database [24] and built a neighbor-joining gene tree of bacterial Hsp90s (Figure S1A–B). We observed two well-supported long-branching clades as well as several less confident divisions in the tree (Figure S1B). These two long-branching clades contain sequences corresponding to the ‘*hsp90B*’ and ‘*hsp90C*’ paralogs that were described previously [11], [12]. All other branches correspond to ‘*hsp90A*’ [11], which is the largest of the Hsp90 families in bacteria (Figure S1C, Text S1). Notably, *hsp90A* is the lineage out of which all eukaryotic Hsp90s (excluding mitochondrial and chloroplast Hsp90s) are derived. Moreover, the *E. coli* gene *htpG* belongs to the *hsp90A* family, and its gene product is the best-studied bacterial Hsp90 protein. For these reasons, we restricted our analysis to *hsp90A*.

### Genome-wide detection of genes co-evolving with *hsp90A*

We set out to identify orthologous groups whose presence and absence profiles across bacterial species are associated with the presence and absence profile of *hsp90A*. To avoid spurious associations, any such comparative analysis must go beyond a naïve comparison of presence/absence patterns across genomes and incorporate phylogenetic information [25]. To this end, we used BayesTraits [26]–[28], a computational framework for phylogenetic analysis of character evolution. Given the states (e.g., presence/absence) of two characters across some set of species and a phylogenetic tree relating these species, BayesTraits evaluates the likelihood of various evolutionary models throughout the tree. This approach can be utilized, for example, to determine whether these two characters evolve in a mutually dependent vs. an independent fashion.

**Figure 1:**
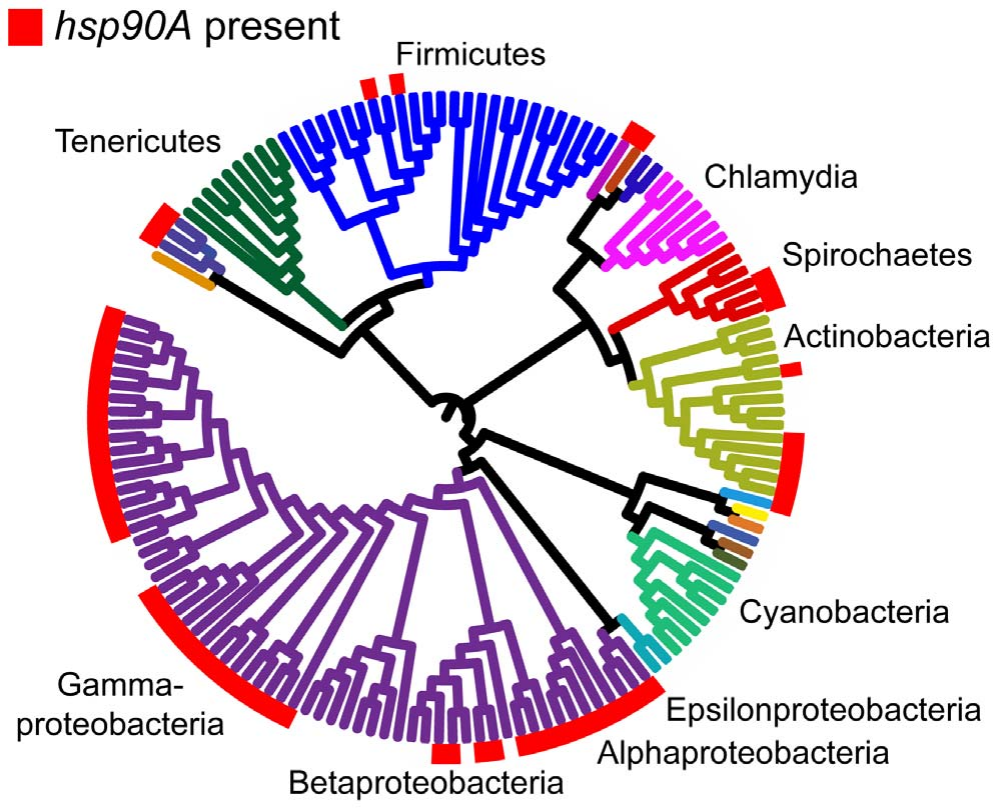
The distribution of *hsp90A* across a bacterial phylogeny. Branches are colored according to phyla. Large taxonomic groups are labeled. Branch lengths are ignored for ease of display. The phylogeny constructed by Ciccarelli *et al.* [29] is used (see Methods). For distribution of other bacterial Hsp90 paralogs, see Figure S1C. *hsp90B* and *hsp90C* are not displayed, and are ignored throughout the analysis.

We used BayesTraits to detect associations between *hsp90A* and 4646 other orthologous groups in bacteria (which hereafter we shall refer to as ‘genes’ for simplicity). We used the tree constructed by Ciccarelli *et al.* [29] as a model phylogeny (Figure 1). In this initial analysis, we tested for any kind of dependency between *hsp90A* and other genes, and did not make specific assumptions about the nature of the relationship between *hsp90A* and the genes in question [28]. Specifically, we compared a model in which the rate of gain and loss of a given gene is independent of the rate of gain and loss of *hsp90A* (independent evolution) vs. a model in which the rate of gain and loss of this gene is affected by the presence or absence of *hsp90A* or vice-versa (co-evolution).

In total, we found 327 genes that co-evolve with *hsp90A* (Dataset S1). We will refer to this set as *hsp90A*-associated genes. These *hsp90A*-associated genes were significantly enriched for annotations related to the flagellum and to bacterial secretion systems (Table 1). Moreover, out of the 16 *hsp90A*-associated bacterial secretion genes, 10 were part of the non-flagellar Type III secretion system, suggesting that *hsp90A* is associated specifically with this system rather than with secretion systems in general. Using a different and markedly more extensive phylogeny [30] provided similar results (see Text S1, Table S1), as did a pruned Ciccarelli tree without the species containing the *hsp90B* or *hsp90C* (see Text S1).

**Table 1:** Functional enrichments in the classes of *hsp90A*-associated genes.

Functional Class (KEGG)	P-Value	Number of genes*
***All genes co-evolving with hsp90A (327 genes)***		
Flagellar assembly [PATHko02040]	9.6E-24	27/39
Bacterial motility proteins [BRko02035]	9.6E-14	35/111
Bacterial chemotaxis [PATHko02030]	8.9E-07	10/26
Bacterial secretion system [PATHko03070]	3.0E-06	16/65
***Genes upon which hsp90A is dependent (70 genes)***		
Bacterial secretion system [PATHko03070]	1.4E-05	6/65
Secretion system [BRko02044]	1.4E-05	11/217
***Genes dependent on hsp90A (139 genes)***		
Flagellar assembly [PATHko02040]	2.8E-28	25/39
Bacterial motility proteins [BRko02035]	5.3E-20	31/111
Bacterial chemotaxis [PATHko02030]	2.6E-07	8/26
***Genes mutually dependent with hsp90A (103 genes)***		
Bacterial secretion system [PATHko03070]	9.1E-08	10/65
Staphylococcus aureus infection [PATHko05150]	4.5E-07	4/9

* **The number of genes with this functional annotation in the** ***hsp90A*** **-associated set and in the background set.**

### Characterization of co-evolutionary dynamics

The associations of *hsp90A* with other genes identified above are agnostic to the specific nature of the dependency between *hsp90A* and the gene in question. For example, our initial analysis could not distinguish between a positive association (i.e. genes tend to be gained and lost together) and a negative association (i.e. genes tend not to co-occur in genomes). Similarly, this analysis did not distinguish between genes whose gains and losses are affected by the presence of *hsp90A* (but that do not themselves affect *hsp90A* evolution) and genes that exhibit mutually dependent dynamics with *hsp90A*. Without a quantitative estimate of the effects that *hsp90A* and its co-evolving partners have upon one another, inference of Hsp90A function and its relationship with other genes is challenging.

**Figure 2:**
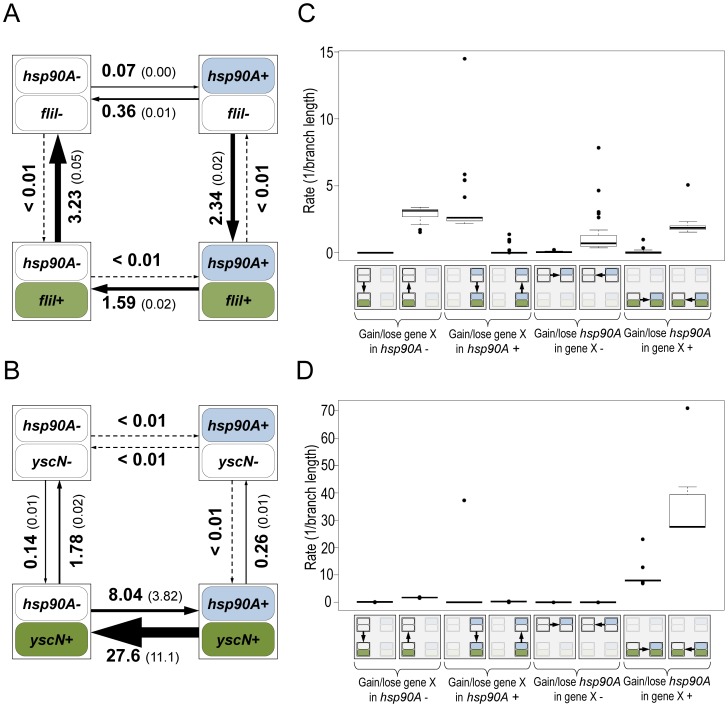
Flagellar genes and secretion system genes show distinct signatures of co-evolution with *hsp90A*. Schematic diagrams of the models describing the co-evolution of *hsp90A* with the flagellar gene *fliI* (A) and the non-flagellar Type III secretion gene *yscN* (B). The four boxes represent the four possible states of presence and absence in each model, and arrows represent transitions between them (gain or loss events). Arrow widths in each diagram are scaled to represent the rate of each transition. The average transition rate and standard deviation across multiple BayesTraits runs are displayed (see Methods). Box plots of the rates of gain and loss of all *hsp90A*-associated flagellar genes (n = 27; **C**) and all *hsp90A*-associated Type III secretion genes (n = 10; **D**) further demonstrate consistent co-evolutionary dynamics of genes in these categories. A box plot of all *hsp90A*-associated secretion genes (including all types) is provided as Figure S4.

To characterize the specific nature of the dependency between *hsp90A* and *hsp90A*-associated genes, we therefore examined rates of gain and loss inferred by BayesTraits. We focused on the two major non-overlapping *hsp90A*-associated functional categories, flagellar assembly and bacterial secretion. Considering, for example, *fliI*, a representative flagellar gene, we found that its gain and loss was strongly affected by the presence of *hsp90A*. Specifically, in the presence of *hsp90A*, *fliI* was often gained and rarely lost, whereas it was rarely gained and often lost when *hsp90A* is absent (Figure 2A). This pattern was common to all *hsp90A*-associated flagellar genes (Figures 2C, S2), suggesting a positive association between *hsp90A* and flagellar genes throughout evolution. In contrast, the co-evolutionary relationship between *hsp90A* and *yscN*, a representative nonflagellar type III secretion system gene, was markedly different, with *yscN* presence strongly affecting the gain and loss of *hsp90A* (Figure 2B). Specifically, the presence of *yscN* was associated with a large increase in the rates of gain and (even more dramatically) loss of *hsp90A* relative to these rates in its absence. Again, this pattern was common to all *hsp90A*-associated bacterial secretion genes (Figures 2D, S3, S4), suggesting a negative association between *hsp90A* and nonflagellar secretion genes throughout evolution.

To further validate the fundamentally distinct co-evolutionary dynamics of these two groups of genes, we considered four different co-evolutionary models: (1) *hsp90A* and the gene in question are independent (null); (2) *hsp90A* and the gene in question are mutually dependent; (3) *hsp90A* is dependent on the gene in question but not vice versa, and (4) the gene in question is dependent upon *hsp90A* but not vice versa (Methods). We used the Akaike Information Criterion (AIC [31]) to determine which of these 4 models best fit the co-evolutionary dynamics of each *hsp90A*-associated gene. As expected, none of the *hsp90A*-associated genes fit the independent model. Of the 27 *hsp90A*-associated flagellar genes, 25 were classified as being dependent on *hsp90A* but not vice-versa (model 4). Of the 16 *hsp90A*-associated secretion system genes, 10 genes were classified as mutually dependent with *hsp90A* (model 2; 6 of which were Type III secretion system genes), whereas 6 were classified as affecting the evolution of *hsp90A* (model 3). Furthermore, considering all *hsp90A*-associated genes, we found that genes that best fit each of the evolutionary dependency models above (models 2, 3, and 4) were enriched for different functions (Table 1). Specifically, among genes dependent on *hsp90A*, flagellar motility was strongly enriched, whereas among genes mutually dependent on *hsp90A*, secretion system components were enriched. Taken together, these patterns suggest that flagellar genes and secretion system genes had markedly different regimes of co-evolution with *hsp90A*.

### Prediction of Hsp90A clients

Although many genes exhibited distinct patterns of co-evolution with *hsp90A*, these patterns could be the result of indirect evolutionary relationships rather than the outcome of a direct interaction with Hsp90A. We therefore aimed to predict specific genes that encode putative *hsp90A* clients. Our method is based on the assumption that strong, conserved clients should be heavily dependent on Hsp90A, and thus should be found only rarely in the absence of *hsp90A* throughout evolution. To estimate the expected frequency of each *hsp90A*-associated gene with and without *hsp90A*, we used the inferred BayesTraits rates to calculate the steady-state probabilities of each of the 4 possible two-gene presence/absence states (Methods). These probabilities represent the proportion of the time that some arbitrary bacterial lineage will spend in each of the presence/absence states throughout evolution. From these probabilities we calculated a *Putative Client Index* (PCI) for each *hsp90A*-associated gene to evaluate how often it was present without *hsp90A* throughout evolution, compared to a null expectation (see Methods). This index is close to zero for genes that were infrequently present without *hsp90A* and were hence likely to be Hsp90A clients. We defined the genes with the lowest PCI values as putative clients (Table 2; see also Text S1).

**Table 2:** Putative Hsp90A clients among 327 *hsp90A*-associated genes.

PCI	KO	Gene product function and KEGG common name
0.041	K03628	transcription termination factor, Rho
0.067	K00074	3-hydroxybutyryl-CoA dehydrogenase, PaaH
0.102	K02427	23S rRNA (uridine2552-2′-O)-methyltransferase, RlmE
0.178	K03770	peptidyl-prolyl cis-trans isomerase D, PpiD
0.203	K06178	23S rRNA pseudouridine2605 synthase, RluB
0.257	K03694	ATP-dependent Clp protease ATP-binding subunit, ClpA
0.269	K01525	bis-nucleosyl tetraphosphate, ApaH
0.295	K07082	UPF0755 protein
0.298	K05788	Integration host factor beta subunit, IhfB
0.299	K15270	S-adenosylmethionine uptake transporter, Sam
0.311	K02411	flagellar assembly protein, FliH
0.341	K02412	flagellum-specific ATP synthase, FliI
0.347	K02417	flagellar motor switch protein, FliN/FliY
0.357	K02419	flagellar biosynthetic protein, FliP
0.358	K02392	flagellar basal-body rod protein, FlgG
0.358	K02388	flagellar basal-body rod protein, FlgC
0.362	K02390	flagellar hook protein, FlgE
0.374	K00795	farnesyl diphosphate synthase, IspA

### Novel and known functions of putative Hsp90 clients

**Figure 3:**
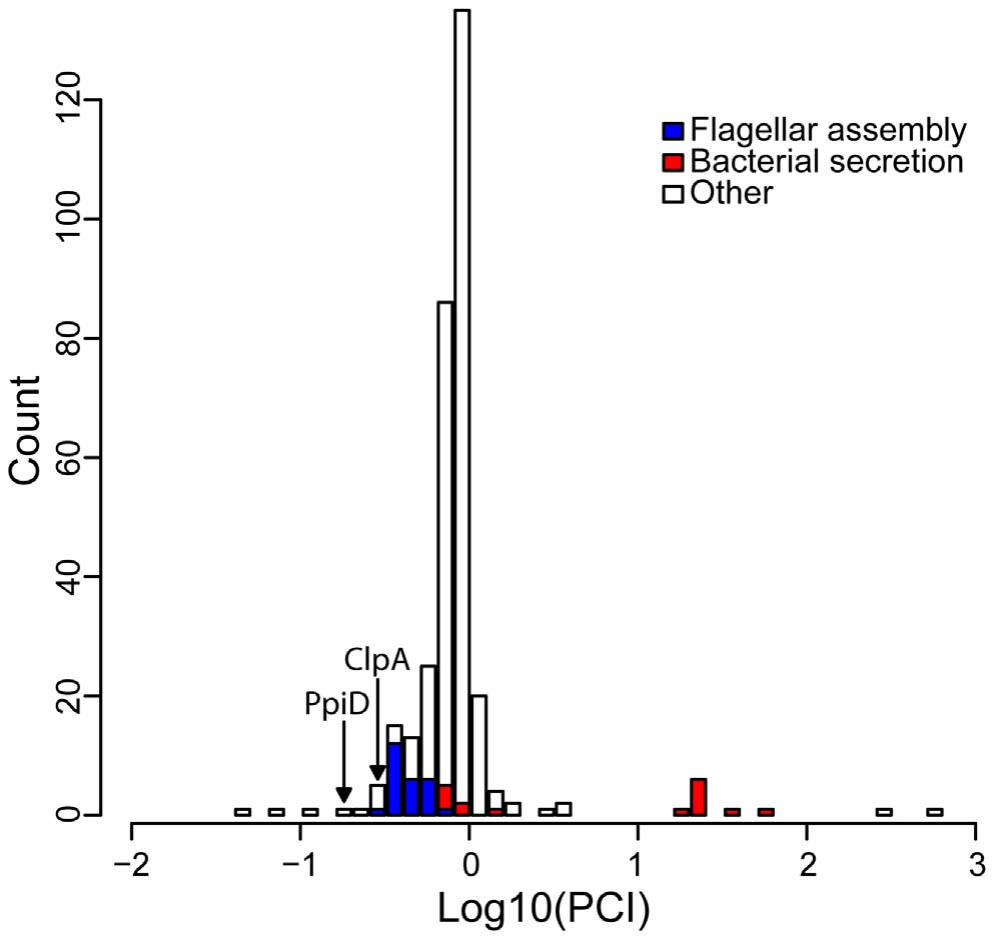
The distribution of the Putative Client Index, *PCI*, among *hsp90A*-associated genes. Lower values indicate behavior closer to that expected of a client. The 18 genes most likely to be clients are listed in Table 2. Prominent functional groups are highlighted, as well as two chaperone-encoding genes.

Consistent with our prior analysis, several flagellar genes behaved as potential clients (Table 2). In particular, our set of putative clients included several genes (*fliH*, *fliI*, *fliN*) whose products had been previously shown to physically interact with Hsp90A in *E. coli* [19]. The products of these genes are cytoplasmic components of the flagellar rotor and export apparatuses. In contrast, nonflagellar type III secretion genes were all absent from the list of potential clients. In fact, nonflagellar type III secretion system components were rated as some of the least likely clients by our index (Figure 3). This disparity in predicted client status mirrors the different evolutionary relationships of these complexes with *hsp90A* (Figure 2).

Chaperone/proteases (e.g. ClpA and PpiD) also ranked high in our list of potential clients. Hsp90A is known to collaborate with other chaperone systems such as DnaK [14], [32] but to date no obligate co-chaperones have been described. The identified chaperone/proteases may represent such co-chaperones or collaborating chaperone systems, since our index cannot discriminate between Hsp90 clients and Hsp90 co-chaperones (or other collaborating proteins). Alternatively, these observed associations could simply indicate that components of the cytoplasmic stress response are dependent upon Hsp90A.

We also found several unexpected putative clients, such as the 3-hydroxybutyryl-CoA dehydrogenase PaaH and the transcription termination factor Rho, which we predict to be the two strongest clients. Further study will be necessary to understand these associations and the underlying cause of the co-evolutionary association between these genes and *hsp90A*.

### Swimming motility and chemotaxis assays of Hsp90A-defective *E. coli*

Our putative clients and the predicted chaperone role of Hsp90A in flagellar assembly are consistent with previous observations. Specifically, the deletion of *E. coli hsp90A*, also known as *htpG*, resulted in reduced surface swarming movement [33]. We also previously observed physical interactions between the HtpG protein and certain flagellar proteins [19]. Yet, these observations lacked a clear demonstration of client status or mechanism, and *E. coli* swarming is a complex behavior that depends on numerous factors in addition to flagellar function [34]. We therefore set out to test our hypothesis that Hsp90A is physiologically important for flagellar assembly and function and that flagellar components are indeed Hsp90A clients.

**Figure 4:**
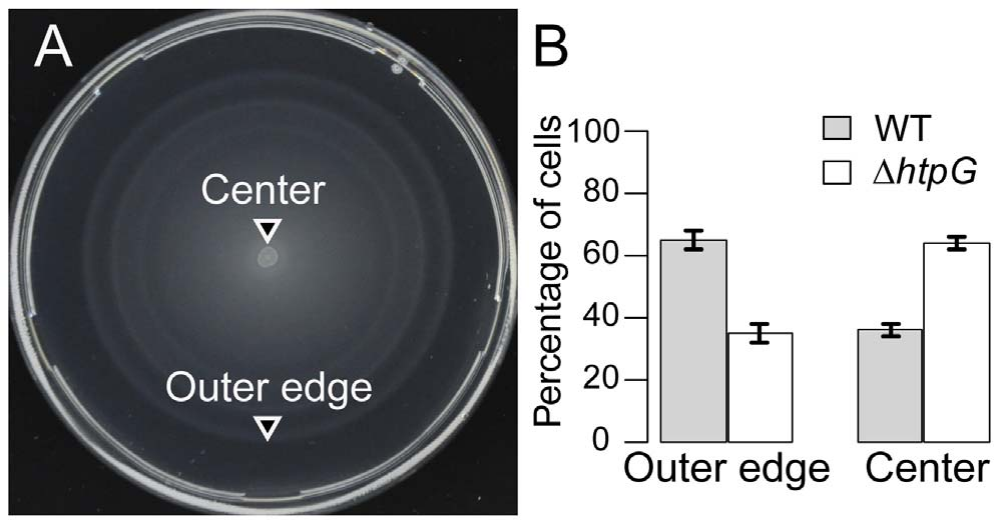
*ΔhtpG E. coli* cells spread less efficiently on soft-agar plates. Upon equal mixing, WT and *ΔhtpG* cells were competed for 8 hours at 34° on the same soft-agar plates, where bacteria spread in a motility- and chemotaxis-dependent fashion. Samples from the outer edge of the plate are thus enriched in cells with optimal chemotaxis and motility, whereas cells from the center are less chemotactic and/or motile. (**A**) A representative image of assay plate. (**B**) Quantitation of different genotypes as determined by percentage of the YFP-labeled WT vs. CFP-labeled *ΔhtpG* cells at the indicated locations. YFP and CFP expression was induced by 1 µM IPTG. An essentially identical result was obtained for the CFP-labeled WT vs. YFP-labeled *ΔhtpG* cells (data not shown), confirming that it is label-independent. Error bars indicate standard errors from four replicates. Results were similar at 42°C (Table S3).

We examined the swimming motility phenotype of *ΔhtpG E. coli* strains on soft-agar plates (Methods). In contrast to surface swarming, swimming is a less complex behavior, in which bacteria use functional flagella and chemotaxis components to swim from an inoculation point through agar pores, following nutrient gradients that are created by nutrient depletion within the colony. The soft-agar assay is routinely used to assay bacterial swimming motility and chemotaxis. To enhance our ability to detect differences between wild-type and *ΔhtpG* cells, the assays were performed competitively. Competitive assays emphasize small differences between strains and reduce experimental error, thereby increasing the sensitivity of the assay. After mixing equal amounts of YFP-labeled WT and CFP-labeled *ΔhtpG* strains, this mixture was inoculated in the center of a soft-agar plate and incubated at 34°C for 8 hrs. We then counted cells of each strain in the plate center vs. the outer edge using fluorescence microscopy (Figure 4A). *ΔhtpG* mutants migrated less efficiently to the plate's outer edge relative to WT, confirming that they are partially deficient in their motility and/or chemotaxis (Figure 4B). This defect is apparently subtle, since little difference between WT and *ΔhtpG* cells was observed in a non-competitive assay (Figure S5), but it could be revealed due to strong selection for cells with optimal motility and chemotaxis at the outer edge of the spreading bacterial population.

We also tested the phenotype of the HtpG(E34A) mutant, which has reduced rates of ATP hydrolysis and is deficient in substrate refolding [14], [35]. Since HtpG ATPase activity is necessary for release of clients, HtpG(E34A) is less efficient at releasing clients [36]–[38]. Indeed, this mutant showed stronger motility/chemotaxis defects than the *ΔhtpG* strain (Figure S5), presumably due to sequestration of its client proteins. We therefore employed the HtpG(E34A) mutant in all subsequent assays as a more sensitive test of HtpG involvement. Taken together, our observations suggest that the motility defect may be due to the improper function or sequestration of HtpG clients.

### FRET observation of HtpG interactions with flagellar motor components

**Figure 5:**
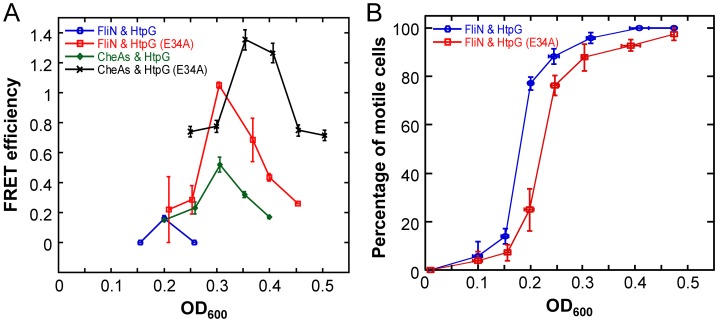
Growth-stage-dependent interaction of HtpG with FliN and CheA. (**A**) Efficiency of FRET between HtpG-YFP or HtpG(E34A)-YFP and FliN-CFP or CFP-CheA as a function of growth stage (indicated by OD_600_ value), measured by acceptor photobleaching in wild-type cells (Methods). Error bars indicate standard errors from three replicates. For these assays, a truncated form of CheA lacking the first 97 amino acids (CheA_s_) was used because this fusion was more stable against spontaneous proteolysis than the fusion to full-length CheA, but showed similar interaction with HtpG (Table S4) (**B**) Growth-stage dependence of motility in cultures used for FRET measurements in (A), assayed as a percentage of motile cells The onset of cell motility is substantially delayed in cells expressing HtpG(E34A). Error bars indicate standard errors from three replicates.

To further investigate the *in vivo* interaction of HtpG with flagellar components, we used *htpG-yfp* and *htpG*(E34A)-*yfp* constructs expressed in WT cells to perform acceptor photobleaching FRET between HtpG and FliN-CFP over an *E. coli* growth curve. Motility of *E. coli* is known to increase at the transition from the early exponential to post-exponential phase of growth [39], and this experimental design enabled us to examine the HtpG-FliN interaction in the context of the flagellar assembly process. If HtpG is indeed involved in the assembly process of these structures, the interaction of HtpG with FliN should correspond temporally to the timing of flagellar assembly. Indeed, we found that the interaction with FliN peaked at OD_600_ = 0.2 (Figure 5A) and correlated well with the onset of cell motility in wild-type cells (Figure 5B). Moreover, the interaction of HtpG(E34A) with FliN was stronger and delayed compared to the binding of wild-type HtpG. Correspondingly, the onset of motility was delayed in cells expressing HtpG(E34A) (Figure 5B). This is consistent with the delayed release of clients by HtpG(E34A), suggesting that HtpG's role in motility derives from a direct involvement in flagellar complex assembly.

Given that both bacterial and eukaryotic Hsp90s are known to collaborate with Hsp70 in refolding proteins [14], [40]–[42], we considered the possibility that this was also the case for bacterial flagellar assembly. We previously showed that some flagellar motor components interact with DnaK, the *E. coli* Hsp70 homolog [19]. Therefore, we repeated the FRET experiments testing for interactions between HtpG or HtpG(E34A) and FliN in a *ΔcbpAΔdnaJ* background. CbpA and DnaJ are DnaK co-chaperones and are essential for DnaK–dependent refolding activity [14]. DnaK should not be able to pass substrates to HtpG in this mutant background. Indeed, we found that FRET interactions with FliN disappear for both HtpG proteins in this background (Figure S6A), suggesting that DnaK-dependent remodeling precedes HtpG action in flagellar complex assembly.

### FRET observation of HtpG interactions with chemoreceptor components

Since a recent high-throughput assay showed kinases to be overrepresented among eukaryotic Hsp90 clients [43], [44], we next examined whether the HtpG-dependent defects in chemotaxis may also be due to defective chemoreceptor kinase activity. Although no chemotaxis proteins were found in our list of the strongest putative clients, we did observe a significant enrichment of these components in the *hsp90A*-associated set (Table 1). We thus tested interactions between six chemoreceptor cluster components and HtpG(E34A) using, as before, acceptor photobleaching FRET (Table S4). We observed a strong interaction of HtpG(E34A) with the chemoreceptor kinase CheA. Our results suggest that the FliN/HtpG and CheA/HtpG interactions are direct and do not depend on other flagellar or chemotaxis proteins, since these interactions are robust to deletion of *flhC*, which ablates expression of all endogenous flagellar and chemotaxis genes (Table S4) [19]. Moreover, the CheA dimerization domain was required for association with HtpG, supporting the hypothesis that HtpG aids oligomerization of its clients [17], [45]. Testing HtpG interactions with other chemotaxis proteins of *E. coli* revealed an additional strong interaction with the dimeric phosphatase CheZ but not with other proteins (Table S4).

We again examined the temporal dynamics of these interactions. Due to the hierarchical order of flagellar and chemotaxis gene expression [39], [46], the assembly of chemoreceptor clusters is delayed compared to the assembly of flagellar motors as non-motile cells transition into motile cells. Indeed, the interaction of HtpG with CheA peaked at OD_600_ = 0.3, after the FliN peak (Figure 5A). Just as for FliN, the interaction of HtpG(E34A) with CheA was stronger and delayed compared to wild-type HtpG, and the HtpG-CheA interaction disappeared in a *ΔcbpAΔdnaJ* background (Figure S6B). Collectively, these findings suggest that HtpG plays an important role in the assembly of both the flagellar motor and chemoreceptor clusters through separate client interactions.

### Association of *hsp90A* with life history traits in bacteria

**Figure 6:**
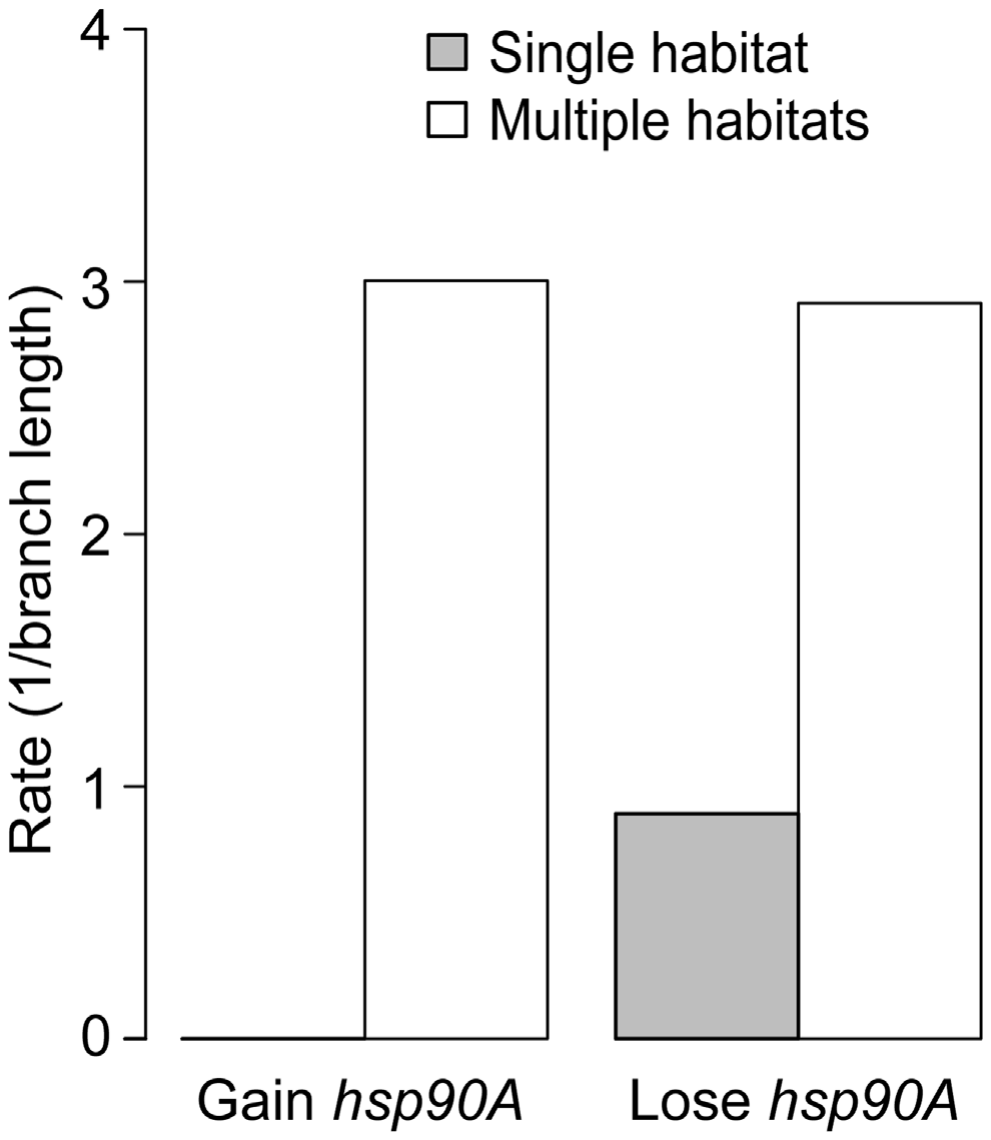
Habitat preference affects the gain and loss of *hsp90A* in bacteria. Rates of gain and loss of *hsp90A* throughout bacterial evolution with relation to multiple habitat preference. Standard deviation across 100 runs was smaller than 0.001 in all cases.

Given the role of HtpG in chaperoning proteins that mediate interactions with the environment, and the known role of eukaryotic Hsp90 in phenotypic robustness, we finally examined whether *hsp90A* directly co-evolved with certain bacterial organismal traits. We considered several organismal traits, including aerobism, thermophilicity, halophilicity, the ability to form endospores, pathogenicity, motility, and habitat preferences (see Methods). We used BayesTraits and the Ciccarelli tree to identify traits that co-evolve with *hsp90A*. Out of the 11 analyzed traits, 4 exhibited significant associations with *hsp90A* (p<0.05; Table S5), with the strongest association observed between *hsp90A* and the capacity to inhabit multiple habitats. Moreover, examining the gain and loss rates obtained, we found that *hsp90A* is gained and lost at significantly higher rates in organisms that inhabit multiple habitats (with no gains inferred in single habitat organisms), suggesting that a preference for multiple habitats imposes a different selection regime on *hsp90A* (Figure 6). We also tested whether the co-evolutionary dependency between *hsp90A* and multiple-habitat preferences was unidirectional, as we observed for some *hsp90A*-associated genes. Comparing the four co-evolutionary models described above and applying AIC to identify the best-fitting model, we found that *hsp90A* gain and loss depended on habitat preference, but not vice versa. This observation suggests that in organisms inhabiting multiple environments *hsp90A* is subjected to dynamically shifting selective pressures, potentially alternating between selection for and against *hsp90A*.

## Discussion

We set out to discover Hsp90 functions conserved throughout the bacterial tree of life. We found that *hsp90A*, the most common paralog of bacterial Hsp90, bore strong signatures of co-evolution with several hundred genes and with specific life history traits, shedding light on its function and impact on evolutionary history. Most notably, we found that *hsp90A* co-evolved with membrane protein complexes such as flagella and other Type III secretion (T3S) systems. Our results suggest that Hsp90's role in sensing and responding to environmental stimuli is conserved between bacteria and eukaryotes.

Similar to verified eukaryotic Hsp90 clients [5], our predicted putative Hsp90A clients were a diverse group of proteins (e.g. the flagella protein FliN, the chaperone ClpA, and the ribosomal protein RluB; see Table 2) that tended to belong to specific functional categories (e.g. flagellar proteins, chaperones, and ribosomal components). As our methods can only infer associations between genes that are frequently gained and lost, we may substantially underestimate the number of *hsp90A*-associated genes and clients. However, the non-essentiality and frequent loss of *hsp90A* throughout bacterial diversity argues that genes not captured in our analysis (since they are not frequently gained and lost) are unlikely to be strongly dependent on the chaperone throughout bacteria.

The subtlety of the bacterial Hsp90 mutant phenotypes that we (and others) report implies that Hsp90's role in cellular physiology has diverged between eukaryotes and prokaryotes [17], [45], [47]. In other words, either essential pieces of cellular physiology changed, or Hsp90 function changed. We favor the first hypothesis, because Hsp90 is well-conserved among bacteria, archaea, and humans at the sequence level [13], and retains a similar quaternary structure [48] and biochemical activity [15], [37], [44]. In contrast, bacterial and archaeal cells differ significantly from eukaryotic cells. Eukaryotic cells have higher cell compartmentalization, longer and multifunctional proteins with multiple domains [49], and increased protein interactome complexity [50]. Together with the existence of many eukaryotic Hsp90 co-chaperones, all these features may contribute to the greater essentiality of Hsp90 in eukaryotes.

The dependence of HtpG-client interactions upon the DnaK chaperone system, as observed by us and by others [14], [15], argues that Hsp90A is well-integrated with other chaperone systems. Our putative clients included ClpA, the substrate adaptor for the ClpAP/ClpAXP chaperone/protease complexes, and PpiD, a periplasmic chaperone [51]. Like HtpG, PpiD is necessary for optimal swarming motility [33], suggesting that it may participate in flagellar assembly. We speculate that these proteins act as Hsp90A co-chaperones in some bacteria; alternatively, their dependence on Hsp90A may represent an example of collaborating chaperone systems.

The best-characterized Hsp90 client in bacteria is the structural ribosomal protein L2 [15], [18], which is near-universally conserved throughout life (and hence not detectable by our method). In addition to L2, other ribosomal proteins were found to interact with HtpG in large-scale proteomics analyses. In agreement with these observations, we found the ribosomal proteins RlmE and RluB among the predicted *hsp90A* clients.

Although these chaperone and ribosomal proteins were predicted to be stronger clients than flagellar proteins, our experimental validation focused on the latter as their client status was suggested by previous observations [19], [33]. We present four lines of evidence for HtpG client status for the flagellar protein FliN and the chemoreceptor kinase CheA, including direct interactions with HtpG, physiologically relevant timing of HtpG-FliN/CheA interactions, phenotypic consequences of reduced HtpG function in CheA/FliN-dependent traits, and dependence of CheA/FliN interactions with HtpG upon the Hsp40-Hsp70 pathway. The identification of FliN and CheA as HtpG clients is consistent with the hypothesis that bacterial Hsp90 facilitates the assembly of large membrane-associated protein complexes [17], [45].

Curiously, whereas the flagellar T3S system contained Hsp90A clients, the nonflagellar T3S system is predicted to have an antagonistic relationship with Hsp90A. Nonflagellar T3S systems and the flagellar T3S systems are closely related (NF-T3SS and F-T3SS) [52], [53]. 9 NF-T3SS components are directly homologous to flagellar components, of which 8 were found to co-evolve with *hsp90A* in our analysis. Yet, these 8 genes are predicted to co-evolve antagonistically with *hsp90A* (Figure 3), whereas their flagellar homologs are mostly predicted to be clients (for instance, the *fliI* and *yscN* genes shown in Figure 2 are homologous). This result suggests that some relationship with Hsp90A is conserved between the two T3S systems, but with apparently opposite effects in each system. This result may reflect the fact that each of these systems is an adaptation to different ecological challenges. Specifically, we have shown that Hsp90A is important for flagellum-enabled motility and chemotaxis in *E. coli*. This mode of motility is strongly adaptive in certain physical environments [34], [54], [55], and thus Hsp90A is likely to be associated with fitness in these environments through flagellar assembly. The presence of NF-T3SS is likewise an adaptation to certain biotic environments [55], [56]. Our observation that organisms inhabiting multiple habitats experience fluctuating selection for *hsp90A* is also consistent with competing selection pressures. Representative genes of these homologous T3S families were not significantly associated with habitat preferences, arguing that *hsp90A*'s association with habitat preferences is not a byproduct of associations with T3S systems. Nonetheless, we suggest that these two T3S systems constitute a link between Hsp90A and phenotypic robustness across different environments.

Inferring function from evolutionary associations has some caveats. For instance, F-T3S systems can be found in genomes that lack *hsp90A*. If F-T3S systems include Hsp90A clients, then what may render Hsp90A-dependent stabilization dispensable in some bacteria? Experimental validation will be necessary to answer such questions, and to distinguish true client relationships from indirect co-evolutionary associations. As discussed before, our method is subject to gene set bias, in that only genes that are gained and/or lost frequently will have enough statistical power to reject the null hypothesis. Similarly, as our method assumes that relationships are maintained throughout the analyzed phylogeny, we cannot reliably detect genes that are associated with *hsp90A* in some organisms but not in others.

Although much work remains to articulate the precise mechanistic relationships between *hsp90*A and its co-evolving genes, our results highlight the tremendous potential of evolutionary inference for guiding experimental research. More generally, our study provides a successful example of how evolutionary perspectives and phylogenetic analyses can inform and advance the study of complex biological systems and the inference of elusive biological functions.

## Methods

### Prokaryotic Hsp90 paralogs

We downloaded all Hsp90 amino acid sequences (including all paralogs) for bacteria with full KEGG genome annotations from the KEGG database [24], [57]. We aligned these sequences using *ClustalO* [58], and used the PHYLIP package [59] to construct neighbor-joining trees and assess their phylogenetic support through bootstrapping. We assigned Hsp90 families to branches according to bootstrap support for the branch and previous classifications [11], [12].

### Genome data

We acquired presence/absence patterns of genes across organisms from the KEGG database release 60.0 (in the form of KEGG Orthology/KO profiles) [57], and functional annotations from KEGG Class. Genes that were either present in fewer than five species or absent in fewer than five species in the tree of interest were dropped from our analysis, as these genes are unlikely to show meaningful signatures of co-evolution by this method.

### Phylogenetic trees

We obtained the tree constructed by Ciccarelli *et al.* (Ciccarelli tree) [29] and pruned it to 148 bacterial species for which KEGG genome data was available. We also obtained the LTP104 version of the 16S/23S rRNA tree from the All-Species Living Tree Project (Yarza tree) [30], [60]. We used ARB [61] to prune this tree to bacterial species for which KEGG genome data was available. We further pruned this tree to omit clades placed paraphyletically at the taxonomic levels of phylum, class, order, and family. This filtered tree included 797 bacterial species. As BayesTraits cannot process trees with zero-length branches, all branch lengths equal to zero were replaced with a negligible branch length (0.00001, approximately an order of magnitude smaller than the next smallest branch length in each tree).

### Organismal trait data

We acquired organismal trait data from the NCBI Entrez genome project, November 2011 [62]. We recoded all traits into presence/absence patterns for the trait in question. For instance, an organism found to be pathogenic towards any other organism was coded as ‘1’ for the trait of pathogenicity, whereas an annotated organism that was never found to be pathogenic was coded as ‘0’. Similarly, we coded both thermophilic and hyperthermophilic organisms as ‘1’ for the trait of thermophilicity, whereas all other annotated organisms were coded as ‘0’; anaerobic organisms were coded as ‘0’ for the trait of aerobicity, whereas all other annotated organisms were recoded as ‘1’. We define as inhabiting multiple habitats any organism that inhabits more than one of NCBI's habitat categories. For BayesTraits analysis, the tree was pruned to include only species annotated for the trait in question (each trait analysis was accordingly performed on a slightly different set of species; see Table S5 for details on species number for each analysis).

### Detecting evolutionary associations with BayesTraits

A complete description of the BayesTraits (v1.0) framework can be found elsewhere [26]. Briefly, consider a character with 2 states, 0 and 1. If a species has 2 such distinct characters, it can occupy 4 possible states: *1*:(0,0), *2*:(0,1), *3*:(1,0), and *4*:(1,1). Specifically, if these 2 characters represent the presence or absence of two genes, *hsp90A* and gene X, these four states correspond to (*hsp90A−*, X−), (*hsp90A+*, X−), (*hsp90A−*, X+), and (*hsp90A+*, X+). Evolution is then the process by which these genes are gained and lost over time. Consider accordingly an evolutionary process where only one character can change state at a time. Such a process can then be described by 8 parameters for the rates of transition per unit time between these 4 states: *Q* = [*q12, q13, q21, q31, q24, q34, q42, q43*], where *qxy* is the rate of transition from state *x* to state *y*. BayesTraits implements this model of evolution as a continuous-time Markov process and estimates each of these rate parameters by maximum-likelihood (ML). We further validated that these ML-based rates are consistent with reversible-jump Markov chain Monte Carlo-derived estimates (Methods; Text S1). This estimation is based on a phylogeny and on the states of the two characters at the tips of the phylogeny. Having estimated these rates, BayesTraits additionally calculates the likelihood of the model based on the character states at the tips of the phylogeny.

We can further compare different models of evolution by forcing certain parameters to be equal. We specifically considered the following 4 models:

1. *hsp90A* and X are independent (*Q*: *q12 = q34, q21 = q43, q13 = q24, q31 = q42*; 4 parameters total)
2. *hsp90A* and X are mutually dependent (No parameter restrictions; 8 parameters total)
3. X depends on *hsp90A* but not vice versa (*Q*: *q12 = q34, q21 = q43*; 6 parameters total)
4. *hsp90A* depends on X but not vice versa (*Q*: *q13 = q24, q31 = q42*; 6 parameters total)

### Identifying *hsp90A*-associated genes

We used *discrete* from the BayesTraits package [26]–[28] to infer associations between *hsp90A* and other bacterial genes and between *hsp90A* and various organismal traits. We first tested for an evolutionary association with *hsp90A* by comparing model **1** to model **2** above with a likelihood ratio test (LRT), as previously described [28]. In our likelihood-ratio tests, the 2Log(LR) approximates a χ^2^ test statistic for rejecting the independent model as a null hypothesis, and is calculated as twice the difference of the log-likelihoods of a co-evolutionary model and a model of evolutionary independence. The set of genes for which model **2** is preferred (i.e., model **1** is rejected as a null hypothesis) have an evolutionary association with *hsp90A*. Since different runs of the BayesTraits maximum likelihood method can potentially produce different parameter values, we repeated this procedure 100 times, each potentially resulting in a different gene set. We validated that these sets are similar and the choice of gene set does not substantially affect downstream analysis (Text S1). Any gene that was found to be associated with *hsp90A* in at least 90 runs was defined as *hsp90A*-associated gene. See Text S1 for more details.

### Reversible-jump Markov chain Monte Carlo analysis

We selected 10 genes at random from the *hsp90A*-associated set and used the BayesTraits implementation of reversible-jump Markov chain Monte Carlo to estimate the rate parameters for their gain and loss in concert with *hsp90A* [63]. For each of these 10 genes, we used an exponential rate prior with mean and variance equal to 30, and ran the chain for 150 million iterations while sampling every 100 iterations. We discarded the first 75 million iterations as burn-in and used the remaining iterations as a posterior distribution of rate parameter estimates. We used Tracer v1.5 [64] and previously described criteria to evaluate chain convergence in this remaining sample [65]. For each rate, we used the median of its posterior distribution in this sample as a point estimate.

### Co-evolutionary model selection

To provide an accurate description of the co-evolutionary dynamics of *hsp90A*-associated genes, we further applied BayesTraits to these genes, estimating the likelihood of each of the four models described above. We identified the best fit model for each gene using the Akaike Information Criterion (AIC) [31], taking into account both the likelihood score and the number of parameters in each model. We again repeated this procedure 100 times and classified a gene into a specific co-evolutionary model only if it fit this same model in at least 90 runs (see Text S1 for more details). This two stage scheme, first identifying associated genes and then selecting a model that best describes their evolutionary relationship with *hsp90A*, provides a more stringent test of co-evolution and supports a simple approach for multiple testing correction.

### Prediction of Hsp90A clients in bacteria

We used BayesTraits-derived evolutionary transition rates under the fully unrestricted model to estimate residence times in specific states (for instance, the proportion of time spent by bacteria in a state where both *hsp90A* and some other gene are present, vs. the time when only the other gene is present) under steady state dynamics. For a given gene, the probability of being in one of the four states, A: (*hsp90A* absent, Gene absent), B: (*hsp90A* present, Gene absent), C: (*hsp90A* absent, Gene present), D: (*hsp90A* present, Gene present) at a very small increment of time *Δ*t after time t is given by:
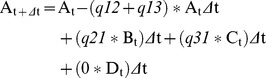


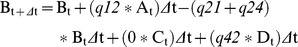


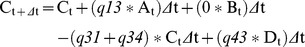


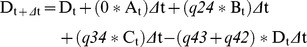
We can differentiate this to obtain the instantaneous change in each probability:
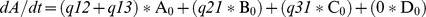


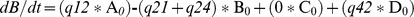


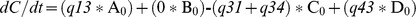


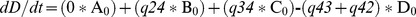
At steady state *dA/dt*  =  0, *dB/dt*  =  0, etc., and therefore:
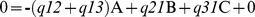


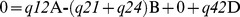


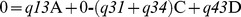


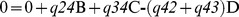
This set of linear equations can be solved for A, B, C, and D, with the requirement that A+B+C+D = 1. We replaced 0 rates with the smallest nonzero rate in the model multiplied by 0.001 to allow transitions between all states. The positive nonzero solution for A, B, C, and D can then be conceived as the expected residence times along some arbitrary bacterial lineage. We used these residence times to estimate a *Putative Client Index*, PCI, denoting the normalized residence time in state C:
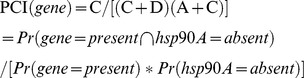
Notably, if Hsp90A and the gene's product have no client relationship, the proportion of time spent in state C is expected to be equal to (C+D)(A+C), so a PCI close to 1 indicates that the observation does not differ from the expectation. Smaller values of PCI therefore indicate that a gene is observed less frequently than expected without *hsp90A*, and is thus more likely to be a client. Since no obvious threshold value can be defined, we considered the 20 genes with the lowest PCI values as putative clients (Figure 3 and Table 2; Methods). To account for variation in rates between BayesTraits runs we repeated this procedure 100 times and defined as putative clients those that were identified as clients in at least 90 of these runs (see Text S1). PCI scores shown in Table 2 and Figure 3 are averages across all runs.

### Functional enrichment analysis

We used a hypergeometric test to assess whether each KEGG Class functional annotation is overrepresented in the various Hsp90-associated gene sets. As a background set in each case we used the entire set of genes analyzed. Any annotation present in less than 4 copies in the background set was not considered. We accepted enrichments at a 5% FDR.

### *E. coli* strains and growth assays

*Escherichia coli* K-12 strains and plasmids used in this study are listed in Table S2. Cells were grown in tryptone broth (TB; 1% tryptone and 0.5% NaCl) and when necessary supplemented with ampicillin, chloramphenicol and/or kanamycin at final concentrations of 100, 35 and 50 µg/ml, respectively. Overnight cultures, grown at 30°C, were diluted 1∶100 and grown at 34°C for about 4 h, to an OD_600_ of 0.45–0.5. All expression constructs for YFP and CFP fusions were constructed as described previously [19], [66], [67]. Induction levels for protein expression were 1 µM IPTG (pHL24, pHL35, pVS129 and pVS132), 20 µM IPTG (pVS64 and pVS99), 25 µM IPTG (pDK36, pDK90 and pDK91), 50 µM IPTG (pDK19 and pVS18), 0.005% arabinose (pHL13, pVS108 and pVS109) and 0.01% arabinose (pHL52, pHL70, pDK14, pDK29, pDK30 and pDK49). Cells were harvested by centrifugation (4,000 rpm, 5 min), washed once with tethering buffer (10 mM potassium phosphate, 0.1 mM EDTA, 1 mM L-methionine, 67 mM sodium chloride, 10 mM sodium lactate, pH 7) and resuspended in 10 mL tethering buffer prior to FRET measurements.

TB soft agar plates were prepared by supplementing TB with 0.3% agar (Applichem) and when necessary with 100 g/mL ampicillin and 1 µM IPTG. Equal amounts of cells from different overnight cultures, adjusted depending on their optical density to the equivalent of 2.5 µl of culture with OD_600_ of 2.0, were inoculated and allowed to spread at indicated temperatures for indicated times. Following incubation, photographs of plates were taken with a Canon EOS 300D (DS6041) camera. Images were analyzed with ImageJ (Wayne Rasband, NIH, http://rsb.info.nih.gov/ij/) to determine the diameter of the rings of spreading colonies.

For analysis of motility at different growth stages (indicated by OD_600_ value), percentages of motile cells were estimated from the microscopy movies of swimming cells. The experiment was performed with the RP437 strain, which is non-motile above 37°C. Cells were grown overnight in TB medium at 37°C to completely inhibit their motility. After dilution in fresh TB medium to OD_600_ 0.01, cells were grown at 34°C for measurements.

### Fluorescence imaging

For microscopy, cells were taken from the soft-agar plates and applied to a thin agarose pad (1% agarose in tethering buffer). Fluorescence imaging was performed on a Zeiss AxioImager microscope equipped with an ORCA AG CCD camera (Hamamatsu), a 100× NA 1.45 objective, and HE YFP (Excitation BP 500/25; Emission BP 535/30) and HE CFP (Excitation BP 436/25; Emission BP 480/40) filter sets. Each imaging experiment was performed in duplicate on independent cultures. All images were acquired under identical conditions. Images were subsequently analysed using ImageJ software.

### Acceptor photobleaching FRET measurement

FRET measurements by acceptor photobleaching were performed on a custom-modified Zeiss Axiovert 200 microscope as described before [66]. Briefly, cells expressing YFP and CFP fusions of interest were concentrated about tenfold by centrifugation, resuspended in tethering buffer and applied to a thin agarose pad (1% agarose in tethering buffer). Excitation light from a 75 XBO lamp, attenuated by a ND60 (0.2) neutral-density filter, passed through a band-pass (BP) 436/20 filter and a 495DCSP dichroic mirror and was reflected on the specimen by a Z440/532 dual-band beamsplitter (transmission 465–500 and 550–640 nm; reflection 425–445 and 532 nm). Bleaching of YFP was accomplished by a 20 sec illumination with a 532 nm diode laser (Rapp OptoElectronic), reflected by the 495DCSP dichroic mirror into the light path. Emission from the field of view, which was narrowed with a diaphragm to the area bleached by the laser, passed through a BP 485/40 filter onto a H7421-40 photon counter (Hamamatsu). For each measurement point, photons were counted over 0.5 s using a counter function of the PCI-6034E board, controlled by a custom-written LabView 7.1 program (both from National Instruments). CFP emission was recorded before and after bleaching of YFP, and FRET was calculated as the CFP signal increase divided by the total signal after bleaching. *ΔflhC* strains were used to define direct interactions between HtpG and flagellar and chemotaxis components. In this background expression of endogenous flagellar and chemotaxis genes is inhibited, thus eliminating indirect interactions that may result from concomitant binding of HtpG and tested protein to a third flagellar or chemotaxis protein.

## Supporting Information

Dataset S1 *hsp90A*-associated genes and relevant information.
(XLS)

Figure S1 Phylogenetic clustering of bacterial *hsp90* paralogs. (**A**) Neighbor-joining phylogeny of 897 bacterial Hsp90 amino acid sequences. Groups Hsp90A, Hsp90B, and Hsp90C as defined by Chen *et al.* [11] are illustrated. (**B**) Consensus neighbor-joining tree for 100 bootstraps with clades collapsed to highlight deep branch structure. Bootstrap support for each branch is displayed and is also reflected by the branch lengths. One species (ZIN, representing Hsp90 from the organism *Candidatus Zinderia insecticola* CARI), never grouped within the other divisions shown, and was excluded from our analysis. The branch separating Hsp90B and Hsp90C from the Hsp90A clades is present in 99/100 bootstrap trees. (**C**) Hsp90A, B, and C presence/absence patterns mapped onto a 16S/23S rRNA phylogeny of 797 bacterial species [30] (see Text S1). Branch lengths are ignored for ease of display.
(TIF)

Figure S2 Co-evolutionary gain and loss rates of all *hsp90A*-associated flagellar genes. The layout of each diagram is similar to that used in Figure 2.
(TIF)

Figure S3 Co-evolutionary gain and loss rates of all *hsp90A*-associated secretion genes. The layout of each diagram is similar to that used in Figure 2.
(TIF)

Figure S4 Box plots of the rates of gain and loss of all *hsp90A*-associated secretion genes (n = 16). See also Figure 2D.
(TIF)

Figure S5 The *htpG*(E34A) mutant strain shows decreased motility/chemotaxis. (**A**) Plates were inoculated with the same amount of wild-type MG1655 (top), the *ΔhtpG* mutant (bottom left) and the *htpG*(E34A) mutant (bottom right) cells and incubated at indicated temperatures for 6 hr. (**B**) Relative motility of *ΔhtpG* and *htpG*(E34A) mutants, compared to wild type, at indicated temperatures, quantified by the diameter of the outer rings of spreading colonies. Error bars indicate standard errors from two replicates.
(TIF)

Figure S6 HtpG interactions with FliN and CheA are dependent on the DnaJ/CbpA/DnaK chaperone system. Acceptor photobleaching FRET was measured between HtpG and FliN (**A**) or CheA (**B**). In each panel, HtpG(E34A) (top row) and wild-type HtpG (bottom row) were assayed, and experiments were performed in both WT (left column) and *ΔdnaJΔcbpA* (right column) backgrounds. Y-axes are normalized in each case to the mean CFP signal before bleaching (first 45 s). Photobleaching begins at ∼50 s and lasts for 20 s (indicated by black bar). FRET interaction is indicated by a post-photobleaching increase in CFP signal above pre-photobleaching CFP signal (as observed in all experiments in the WT background).
(TIF)

Table S1 Comparable results in Ciccarelli and Yarza trees across FDR thresholds.
(DOC)

Table S2 *E. coli* strains and plasmids used in this study.
(DOC)

Table S3 Spreading of wild-type and *ΔhtpG* cells in soft-agar assays at 34°C and 42°C.
(DOC)

Table S4 Acceptor photobleaching FRET interactions of chemotaxis components with HtpG(E34A).
(DOC)

Table S5 *hsp90A* presence and absence is associated with organismal traits in bacteria.
(DOC)

Text S1 Additional details on Hsp90 paralog distribution, consistency of BayesTraits runs, and robustness of co-evolutionary associations to choice of phylogeny.
(DOC)

## References

[1] Rutherford SL, Zuker CS (1994) Protein Folding and the Regulation of Signaling Pathways. Cell 79: 1129–1132. 8001149 10.1016/0092-8674(94)90003-5

[2] Picard D (2002) Heat-shock protein 90, a chaperone for folding and regulation. Cellular and Molecular Life Sciences 59: 1640–1648 doi:10.1007/PL00012491 12475174 10.1007/PL00012491 PMC11337538

[3] Young JC (2001) Hsp90: a specialized but essential protein-folding tool. The Journal of Cell Biology 154: 267–274 doi:10.1083/jcb.200104079 11470816 10.1083/jcb.200104079 PMC2150759

[4] Zhao R, Davey M, Hsu Y-C, Kaplanek P, Tong A, et al (2005) Navigating the chaperone network: an integrative map of physical and genetic interactions mediated by the hsp90 chaperone. Cell 120: 715–727 doi:10.1016/j.cell.2004.12.024 15766533 10.1016/j.cell.2004.12.024

[5] Taipale M, Jarosz DF, Lindquist S (2010) HSP90 at the hub of protein homeostasis: emerging mechanistic insights. Nature reviews Molecular cell biology 11: 515–528 doi:10.1038/nrm2918 20531426 10.1038/nrm2918

[6] Rutherford SL, Lindquist S (1998) Hsp90 as a capacitor for morphological evolution. Nature 396: 336–342 doi:10.1038/24550 9845070 10.1038/24550

[7] Queitsch C, Sangster T a, Lindquist S (2002) Hsp90 as a capacitor of phenotypic variation. Nature 417: 618–624 doi:10.1038/nature749 12050657 10.1038/nature749

[8] Cowen LE, Lindquist S (2005) Hsp90 potentiates the rapid evolution of new traits: drug resistance in diverse fungi. Science 309: 2185–2189 doi:10.1126/science.1118370 16195452 10.1126/science.1118370

[9] Yeyati PL, Bancewicz RM, Maule J, Van Heyningen V (2007) Hsp90 selectively modulates phenotype in vertebrate development. PLoS Genetics 3: e43 doi:10.1371/journal.pgen.0030043 17397257 10.1371/journal.pgen.0030043 PMC1839141

[10] Bardwell JC, Craig E a (1988) Ancient heat shock gene is dispensable. Journal of bacteriology 170: 2977–2983. 3290192 10.1128/jb.170.7.2977-2983.1988 PMC211237

[11] Chen B, Zhong D, Monteiro A (2006) Comparative genomics and evolution of the HSP90 family of genes across all kingdoms of organisms. BMC Genomics 7: 156 doi:10.1186/1471-2164-7-156 16780600 10.1186/1471-2164-7-156 PMC1525184

[12] Stechmann A, Cavalier-Smith T (2004) Evolutionary origins of Hsp90 chaperones and a deep paralogy in their bacterial ancestors. J Eukaryot Microbiol 51: 364–373. 15218707 10.1111/j.1550-7408.2004.tb00580.x

[13] Bardwell JC, Craig E a (1987) Eukaryotic Mr 83,000 heat shock protein has a homologue in Escherichia coli. Proceedings of the National Academy of Sciences of the United States of America 84: 5177–5181. 3299380 10.1073/pnas.84.15.5177 PMC298817

[14] Genest O, Hoskins JR, Camberg JL, Doyle SM, Wickner S (2011) Heat shock protein 90 from Escherichia coli collaborates with the DnaK chaperone system in client protein remodeling. Proceedings of the National Academy of Sciences 108: 8206–8211 doi:10.1073/pnas.1104703108/-/DCSupplemental.www.pnas.org/cgi/doi/10.1073/pnas.1104703108 10.1073/pnas.1104703108 PMC3100916 21525416

[15] Genest O, Reidy M, Street TO, Hoskins JR, Camberg JL, et al (2013) Uncovering a region of heat shock protein 90 important for client binding in E. coli and chaperone function in yeast. Molecular cell 49: 464–473 doi:10.1016/j.molcel.2012.11.017 23260660 10.1016/j.molcel.2012.11.017 PMC3570620

[16] Yosef I, Goren MG, Kiro R, Edgar R, Qimron U (2011) High-temperature protein G is essential for activity of the Escherichia coli clustered regularly interspaced short palindromic repeats (CRISPR)/Cas system. Proceedings of the National Academy of Sciences of the United States of America 108: 20136–20141 doi:10.1073/pnas.1113519108 22114197 10.1073/pnas.1113519108 PMC3250196

[17] Sato T, Minagawa S, Kojima E, Okamoto N, Nakamoto H (2010) HtpG, the prokaryotic homologue of Hsp90, stabilizes a phycobilisome protein in the cyanobacterium Synechococcus elongatus PCC 7942. Molecular microbiology 76: 576–589 doi:10.1111/j.1365-2958.2010.07139.x 20345653 10.1111/j.1365-2958.2010.07139.x

[18] Motojima-Miyazaki Y, Yoshida M, Motojima F (2010) Ribosomal protein L2 associates with E. coli HtpG and activates its ATPase activity. Biochemical and biophysical research communications 400: 241–245 doi:10.1016/j.bbrc.2010.08.047 20727857 10.1016/j.bbrc.2010.08.047

[19] Li H, Sourjik V (2011) Assembly and stability of flagellar motor in Escherichia coli. Molecular Microbiology 80: 886–899 doi:10.1111/j.1365-2958.2011.07557.x 21244534 10.1111/j.1365-2958.2011.07557.x

[20] Peregrín-Alvarez JM, Xiong X, Su C, Parkinson J (2009) The Modular Organization of Protein Interactions in Escherichia coli. PLoS computational biology 5: e1000523 doi:10.1371/journal.pcbi.1000523 19798435 10.1371/journal.pcbi.1000523 PMC2739439

[21] Makhnevych T, Houry WA (2012) The role of Hsp90 in protein complex assembly. Biochimica et biophysica acta 1823: 674–682 doi:10.1016/j.bbamcr.2011.09.001 21945180 10.1016/j.bbamcr.2011.09.001

[22] Hwang S, Rhee SY, Marcotte EM, Lee I (2011) Systematic prediction of gene function in Arabidopsis thaliana using a probabilistic functional gene network. Nature protocols 6: 1429–1442 doi:10.1038/nprot.2011.372 21886106 10.1038/nprot.2011.372 PMC3654671

[23] Wang PI, Hwang S, Kincaid RP, Sullivan CS, Lee I, et al (2012) RIDDLE: Reflective diffusion and local extension reveal functional associations for unannotated gene sets via proximity in a gene network. Genome Biology 13: R125 doi:10.1186/gb-2012-13-12-r125 23268829 10.1186/gb-2012-13-12-r125 PMC4056375

[24] Ogata H, Goto S, Sato K, Fujibuchi W, Bono H, et al (1999) KEGG: Kyoto Encyclopedia of Genes and Genomes. Nucleic acids research 27: 29–34. 9847135 10.1093/nar/27.1.29 PMC148090

[25] Felsenstein J (1985) Phylogenies and the Comparative Method. American Naturalist 125: 1–15.

[26] Pagel M (1994) Detecting Correlated Evolution on Phylogenies: A General Method for the Comparative Analysis of Discrete Characters. Proceedings of the Royal Society B: Biological Sciences 255: 37–45 doi:10.1098/rspb.1994.0006

[27] Barker D, Meade A, Pagel M (2007) Constrained models of evolution lead to improved prediction of functional linkage from correlated gain and loss of genes. Bioinformatics (Oxford, England) 23: 14–20 doi:10.1093/bioinformatics/btl558 10.1093/bioinformatics/btl558 17090580

[28] Barker D, Pagel M (2005) Predicting functional gene links from phylogenetic-statistical analyses of whole genomes. PLoS Computational Biology 1: e3 doi:10.1371/journal.pcbi.0010003 16103904 10.1371/journal.pcbi.0010003 PMC1183509

[29] Ciccarelli FD, Doerks T, Von Mering C, Creevey CJ, Snel B, et al (2006) Toward automatic reconstruction of a highly resolved tree of life. Science 311: 1283–1287 doi:10.1126/science.1123061 16513982 10.1126/science.1123061

[30] Munoz R, Yarza P, Ludwig W, Euzéby J, Amann R, et al (2011) Release LTPs104 of the All-Species Living Tree. Systematic and applied microbiology 34: 169–170 doi:10.1016/j.syapm.2011.03.001 21497273 10.1016/j.syapm.2011.03.001

[31] Akaike HAI (1974) A New Look at the Statistical Model Identification. IEEE Transactions on Automatic Control 19: 716–723.

[32] Kumar M, Sourjik V (2012) Physical map and dynamics of the chaperone network in Escherichia coli. Molecular microbiology 84: 736–747 doi:10.1111/j.1365-2958.2012.08054.x 22463727 10.1111/j.1365-2958.2012.08054.x

[33] Inoue T, Shingaki R, Hirose S, Waki K, Mori H, et al (2007) Genome-wide screening of genes required for swarming motility in Escherichia coli K-12. Journal of bacteriology 189: 950–957 doi:10.1128/JB.01294-06 17122336 10.1128/JB.01294-06 PMC1797309

[34] Partridge JD, Harshey RM (2013) Swarming: flexible roaming plans. Journal of bacteriology 195: 909–918 doi:10.1128/JB.02063-12 23264580 10.1128/JB.02063-12 PMC3571328

[35] Graf C, Stankiewicz M, Kramer G, Mayer MP (2009) Spatially and kinetically resolved changes in the conformational dynamics of the Hsp90 chaperone machine. The EMBO journal 28: 602–613 doi:10.1038/emboj.2008.306 19165152 10.1038/emboj.2008.306 PMC2657576

[36] Panaretou B, Prodromou C, Roe SM, O'Brien R, Ladbury JE, et al (1998) ATP binding and hydrolysis are essential to the function of the Hsp90 molecular chaperone in vivo. The EMBO journal 17: 4829–4836 doi:10.1093/emboj/17.16.4829 9707442 10.1093/emboj/17.16.4829 PMC1170812

[37] Street TO, Lavery LA, Agard DA (2011) Substrate binding drives large-scale conformational changes in the Hsp90 molecular chaperone. Molecular cell 42: 96–105 doi:10.1016/j.molcel.2011.01.029 21474071 10.1016/j.molcel.2011.01.029 PMC3105473

[38] Young JC, Hartl FU (2000) Polypeptide release by Hsp90 involves ATP hydrolysis and is enhanced by the co-chaperone p23. The EMBO journal 19: 5930–5940 doi:10.1093/emboj/19.21.5930 11060043 10.1093/emboj/19.21.5930 PMC305790

[39] Kalir S, McClure J, Pabbaraju K, Southward C, Ronen M, et al (2001) Ordering genes in a flagella pathway by analysis of expression kinetics from living bacteria. Science 292: 2080–2083 doi:10.1126/science.1058758 11408658 10.1126/science.1058758

[40] Schröder H, Langer T, Hartl FU, Bukau B (1993) DnaK, DnaJ and GrpE form a cellular chaperone machinery capable of repairing heat-induced protein damage. the The EMBO Journal 12: 4137–4144. 7900997 10.1002/j.1460-2075.1993.tb06097.x PMC413706

[41] Szabo A, Langer T, Schröder H, Flanagan J, Bukau B, et al (1994) The ATP hydrolysis-dependent reaction cycle of the Escherichia coli Hsp70 system DnaK, DnaJ, and GrpE. Proceedings of the National Academy of Sciences of the United States of America 91: 10345–10349. 7937953 10.1073/pnas.91.22.10345 PMC45016

[42] Herbst R, Gast K, Seckler R (1998) Folding of firefly (Photinus pyralis) luciferase: aggregation and reactivation of unfolding intermediates. Biochemistry 37: 6586–6597 doi:10.1021/bi972928i 9572876 10.1021/bi972928i

[43] Taipale M, Krykbaeva I, Koeva M, Kayatekin C, Westover KD, et al (2012) Quantitative analysis of HSP90-client interactions reveals principles of substrate recognition. Cell 150: 987–1001 doi:10.1016/j.cell.2012.06.047 22939624 10.1016/j.cell.2012.06.047 PMC3894786

[44] Röhl A, Rohrberg J, Buchner J (2013) The chaperone Hsp90: changing partners for demanding clients. Trends in biochemical sciences 38 5: 253–262 doi:10.1016/j.tibs.2013.02.003 23507089 10.1016/j.tibs.2013.02.003

[45] Buchner J (2010) Bacterial Hsp90–desperately seeking clients. Molecular microbiology 76: 540–544 doi:10.1111/j.1365-2958.2010.07140.x 20345652 10.1111/j.1365-2958.2010.07140.x

[46] Chevance FF V, Hughes KT (2008) Coordinating assembly of a bacterial macromolecular machine. Nature reviews Microbiology 6: 455–465 doi:10.1038/nrmicro1887 18483484 10.1038/nrmicro1887 PMC5963726

[47] Richter K, Buchner J (2011) Closing in on the Hsp90 chaperone-client relationship. Structure 19: 445–446 doi:10.1016/j.str.2011.03.007 21481768 10.1016/j.str.2011.03.007

[48] Southworth DR, Agard DA (2008) Species-dependent ensembles of conserved conformational states define the Hsp90 chaperone ATPase cycle. Molecular cell 32: 631–640 doi:10.1016/j.molcel.2008.10.024 19061638 10.1016/j.molcel.2008.10.024 PMC2633443

[49] Brocchieri L, Karlin S (2005) Protein length in eukaryotic and prokaryotic proteomes. Nucleic acids research 33: 3390–3400 doi:10.1093/nar/gki615 15951512 10.1093/nar/gki615 PMC1150220

[50] Fernández A, Lynch M (2011) Non-adaptive origins of interactome complexity. Nature 474: 502–505 doi:10.1038/nature09992 21593762 10.1038/nature09992 PMC3121905

[51] Dartigalongue C, Raina S (1998) A new heat-shock gene, ppiD, encodes a peptidyl-prolyl isomerase required for folding of outer membrane proteins in Escherichia coli. The EMBO journal 17: 3968–3980 doi:10.1093/emboj/17.14.3968 9670013 10.1093/emboj/17.14.3968 PMC1170731

[52] Desvaux M, Hébraud M, Henderson IR, Pallen MJ (2006) Type III secretion: what's in a name? Trends in microbiology 14: 157–160 doi:10.1016/j.tim.2006.02.009 16533600 10.1016/j.tim.2006.02.009

[53] Abby SS, Rocha EPC (2012) The Non-Flagellar Type III Secretion System Evolved from the Bacterial Flagellum and Diversified into Host-Cell Adapted Systems. PLoS Genetics 8: e1002983 doi:10.1371/journal.pgen.1002983 23028376 10.1371/journal.pgen.1002983 PMC3459982

[54] Fraser GM, Hughes C (1999) Swarming motility. Current opinion in microbiology 2: 630–635. 10607626 10.1016/s1369-5274(99)00033-8

[55] Wei Y, Wang X, Liu J, Nememan I, Singh AH, et al (2011) The population dynamics of bacteria in physically structured habitats and the adaptive virtue of random motility. Proceedings of the National Academy of Sciences of the United States of America 108: 4047–4052 doi:10.1073/pnas.1013499108 21325053 10.1073/pnas.1013499108 PMC3053974

[56] Jousset A (2012) Ecological and evolutive implications of bacterial defences against predators. Environmental microbiology 14: 1830–1843 doi:10.1111/j.1462-2920.2011.02627.x 22040156 10.1111/j.1462-2920.2011.02627.x

[57] Tanabe M, Kanehisa M (2012) Using the KEGG database resource. Current protocols in bioinformatics/editoral board, Andreas D Baxevanis. [et al] Chapter 1: Unit1.12 doi:10.1002/0471250953.bi0112s38 10.1002/0471250953.bi0112s38 22700311

[58] Sievers F, Wilm A, Dineen D, Gibson TJ, Karplus K, et al (2011) Fast, scalable generation of high-quality protein multiple sequence alignments using Clustal Omega. Molecular systems biology 7: 539 doi:10.1038/msb.2011.75 21988835 10.1038/msb.2011.75 PMC3261699

[59] Felsenstein J (1989) PHYLIP - Phylogeny inference package (Version 3.2). Cladistics 5: 164–166.

[60] Yarza P, Richter M, Peplies J, Euzeby J, Amann R, et al (2008) The All-Species Living Tree project: a 16S rRNA-based phylogenetic tree of all sequenced type strains. Systematic and applied microbiology 31: 241–250 doi:10.1016/j.syapm.2008.07.001 18692976 10.1016/j.syapm.2008.07.001

[61] Ludwig W, Strunk O, Westram R, Richter L, Meier H, et al (2004) ARB: a software environment for sequence data. Nucleic acids research 32: 1363–1371 doi:10.1093/nar/gkh293 14985472 10.1093/nar/gkh293 PMC390282

[62] http://www.ncbi.nlm.nih.gov/genomes/lproks.cgi.

[63] Pagel M, Meade A (2006) Bayesian analysis of correlated evolution of discrete characters by reversible-jump Markov chain Monte Carlo. The American naturalist 167: 808–825. 10.1086/503444 16685633

[64] Rambaut A, Drummond A (2008) Tracer v1.5, Available from http://beast.bio.ed.ac.uk/Tracer.

[65] Shultz S, Opie C, Atkinson QD (2011) Stepwise evolution of stable sociality in primates. Nature 479: 219–222 doi:10.1038/nature10601 22071768 10.1038/nature10601

[66] Kentner D, Sourjik V (2009) Dynamic map of protein interactions in the Escherichia coli chemotaxis pathway. Molecular Systems Biology 5: 238. 19156130 10.1038/msb.2008.77 PMC2644175

[67] Sourjik V, Berg HC (2000) Localization of components of the chemotaxis machinery of Escherichia coli using fluorescent protein fusions. Molecular Microbiology 37: 740–751. 10972797 10.1046/j.1365-2958.2000.02044.x

