# Supplementary figures and images for: Genome-scale Co-evolutionary Inference Identifies Functions and Clients of Bacterial Hsp90
Source: PLoS Genet. 2013 Jul 11;9(7):e1003631. doi: 10.1371/journal.pgen.1003631 (PMC3708813; doi:10.1371/journal.pgen.1003631)

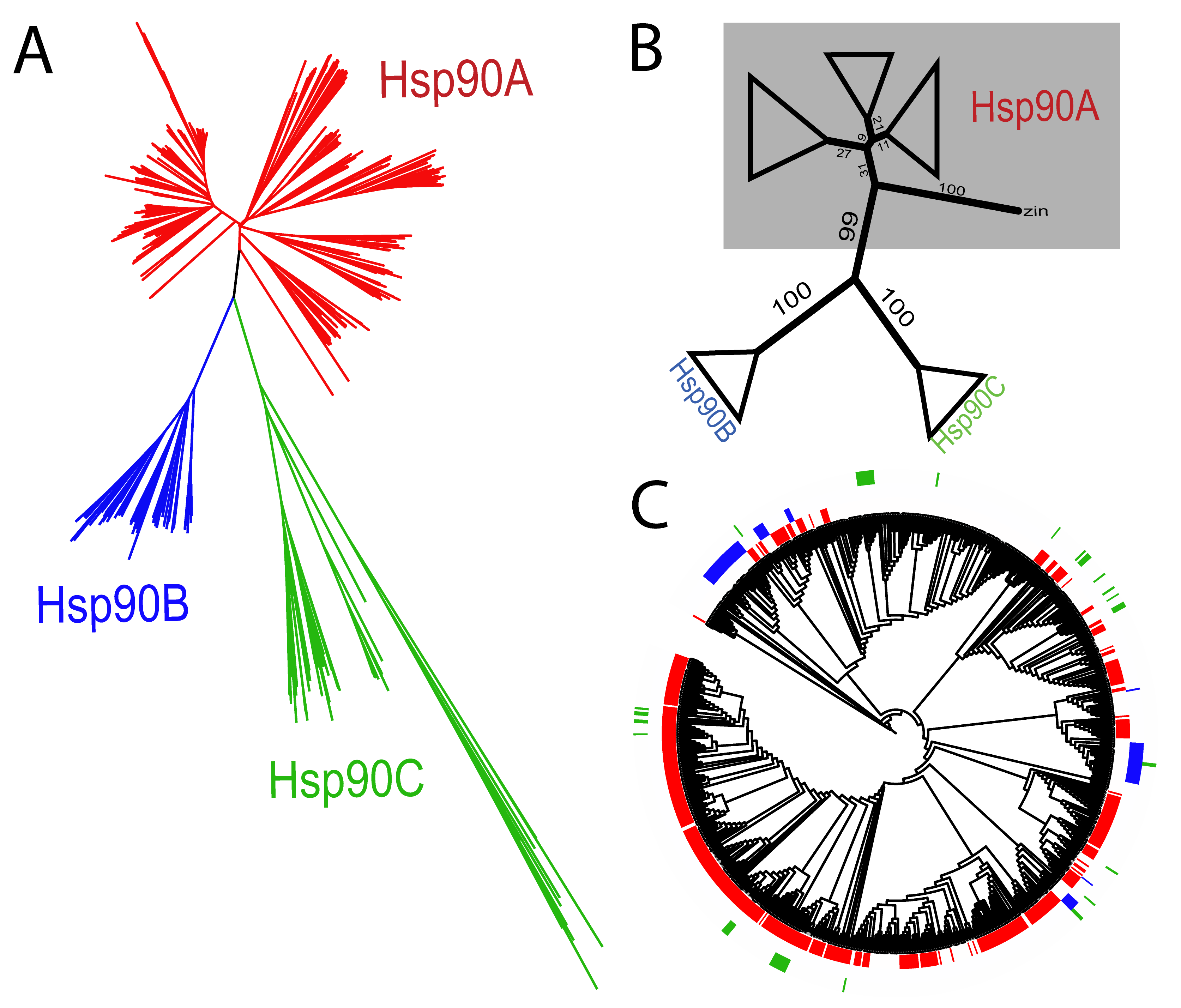

Supplement: Figure S1 — Phylogenetic clustering of bacterial hsp90 paralogs. (A) Neighbor-joining phylogeny of 897 bacterial Hsp90 amino acid sequences. Groups Hsp90A, Hsp90B, and Hsp90C as defined by Chen et al. [11] are illustrated. (B) Consensus neighbor-joining tree for 100 bootstraps with clades collapsed to highlight deep branch structure. Bootstrap support for each branch is displayed and is also reflected by the branch lengths. One species (ZIN, representing Hsp90 from the organism Candidatus Zinderia insecticola CARI), never grouped within the other divisions shown, and was excluded from our analysis. The branch separating Hsp90B and Hsp90C from the Hsp90A clades is present in 99/100 bootstrap trees. (C) Hsp90A, B, and C presence/absence patterns mapped onto a 16S/23S rRNA phylogeny of 797 bacterial species [30] (see Text S1). Branch lengths are ignored for ease of display. (TIF) [file pgen.1003631.s002.tif]

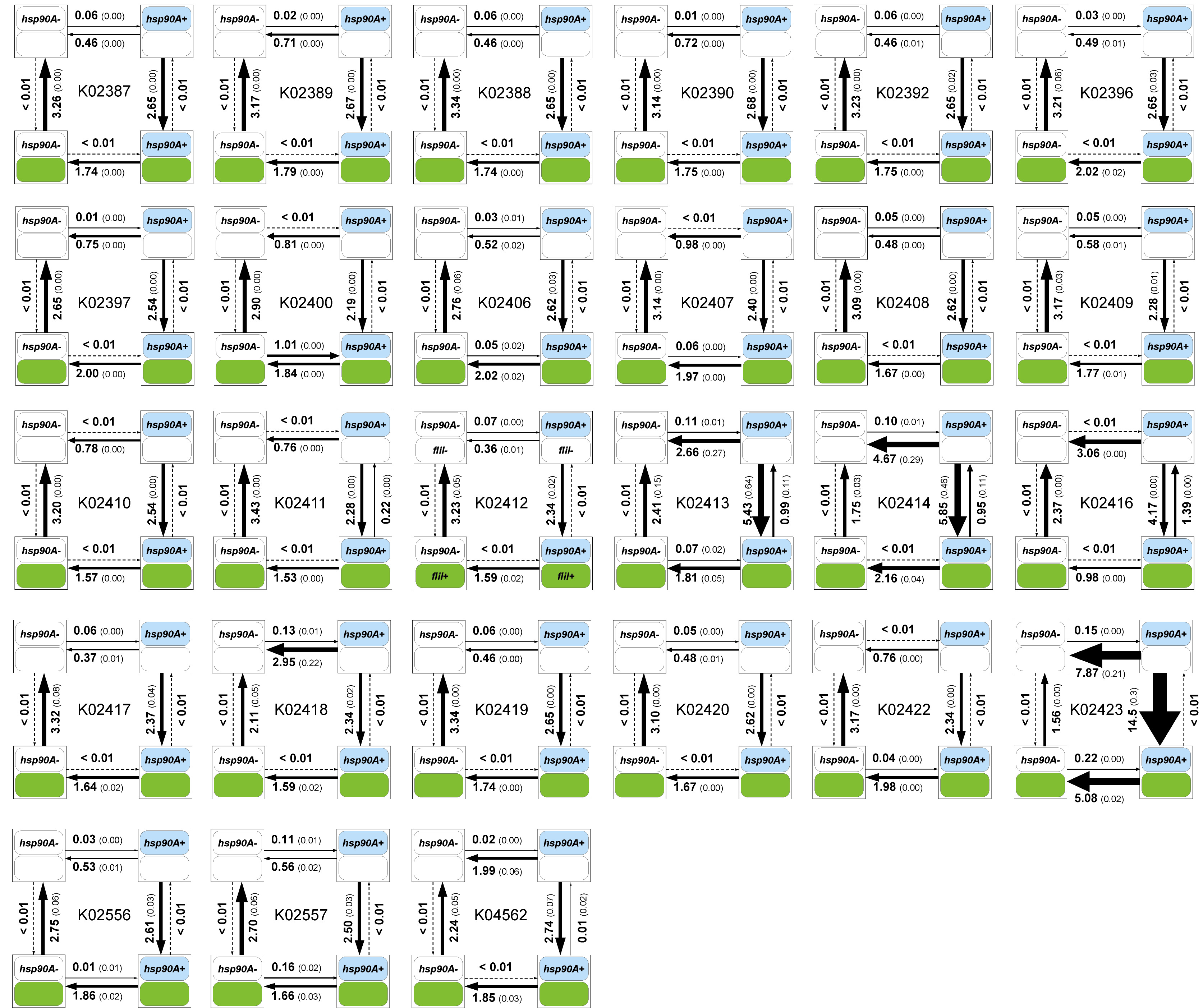

Supplement: Figure S2 — Co-evolutionary gain and loss rates of all hsp90A-associated flagellar genes. The layout of each diagram is similar to that used in Figure 2. (TIF) [file pgen.1003631.s003.tif]

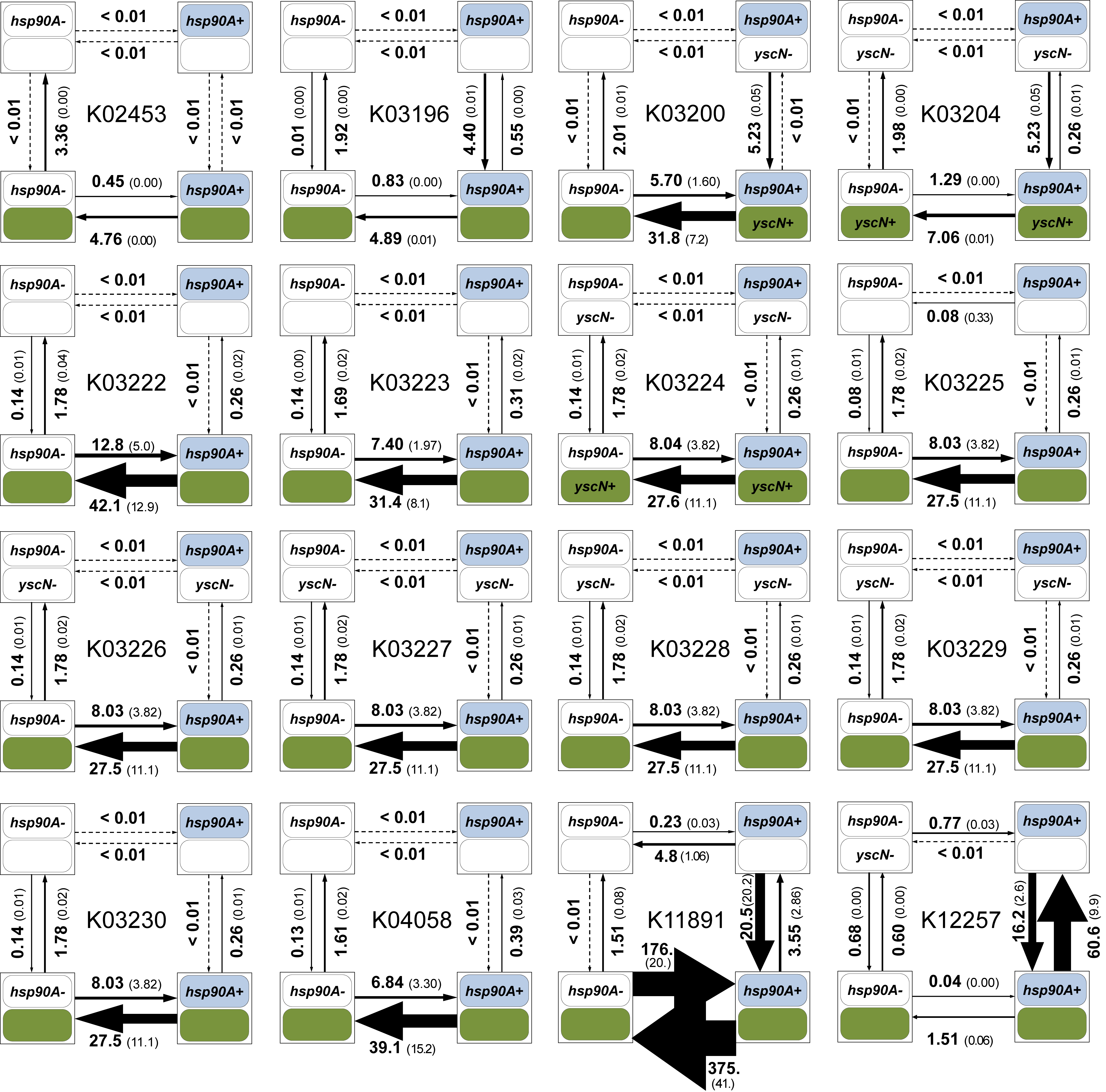

Supplement: Figure S3 — Co-evolutionary gain and loss rates of all hsp90A-associated secretion genes. The layout of each diagram is similar to that used in Figure 2. (TIF) [file pgen.1003631.s004.tif]

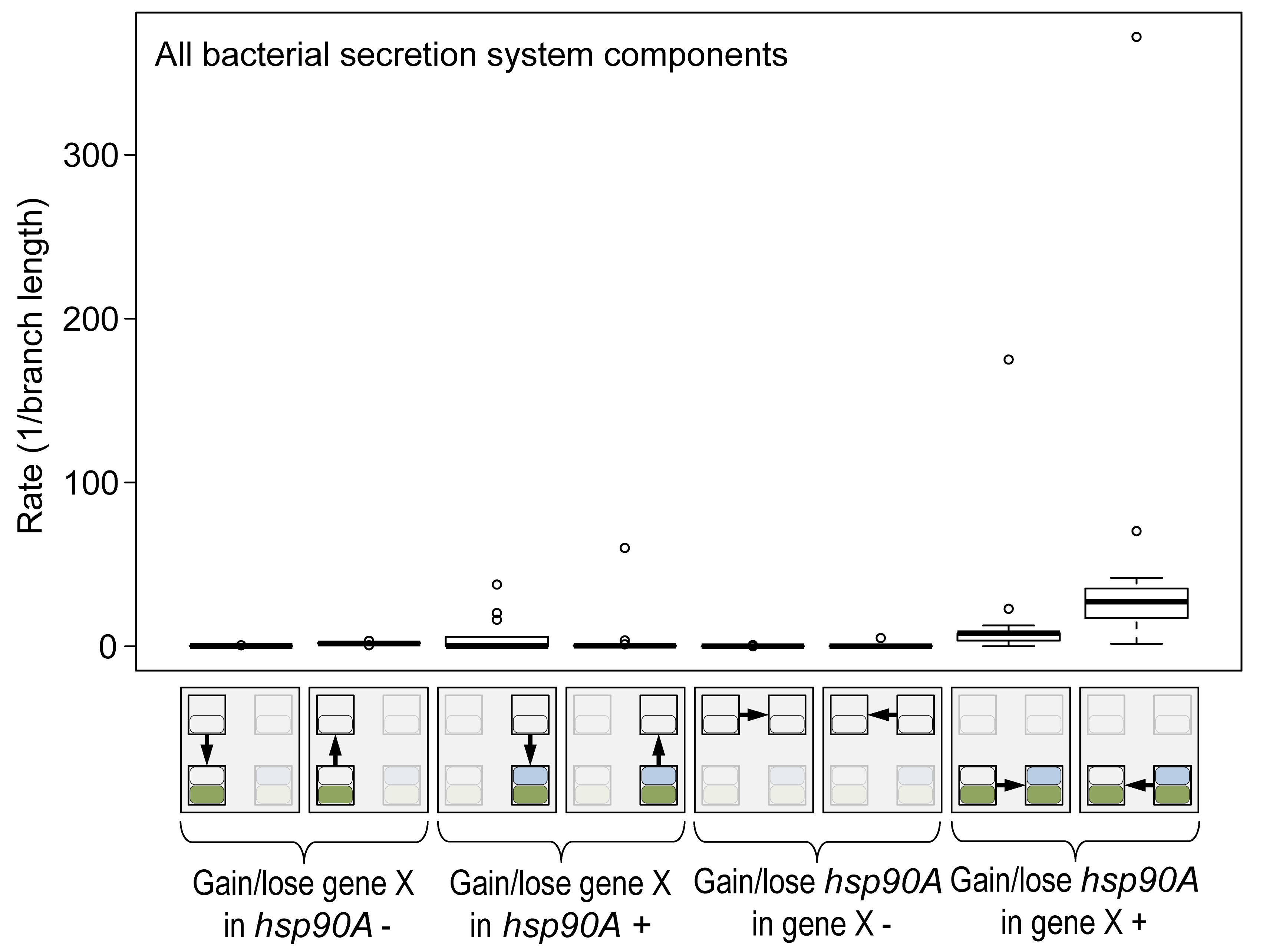

Supplement: Figure S4 — Box plots of the rates of gain and loss of all hsp90A-associated secretion genes (n = 16). See also Figure 2D. (TIF) [file pgen.1003631.s005.tif]

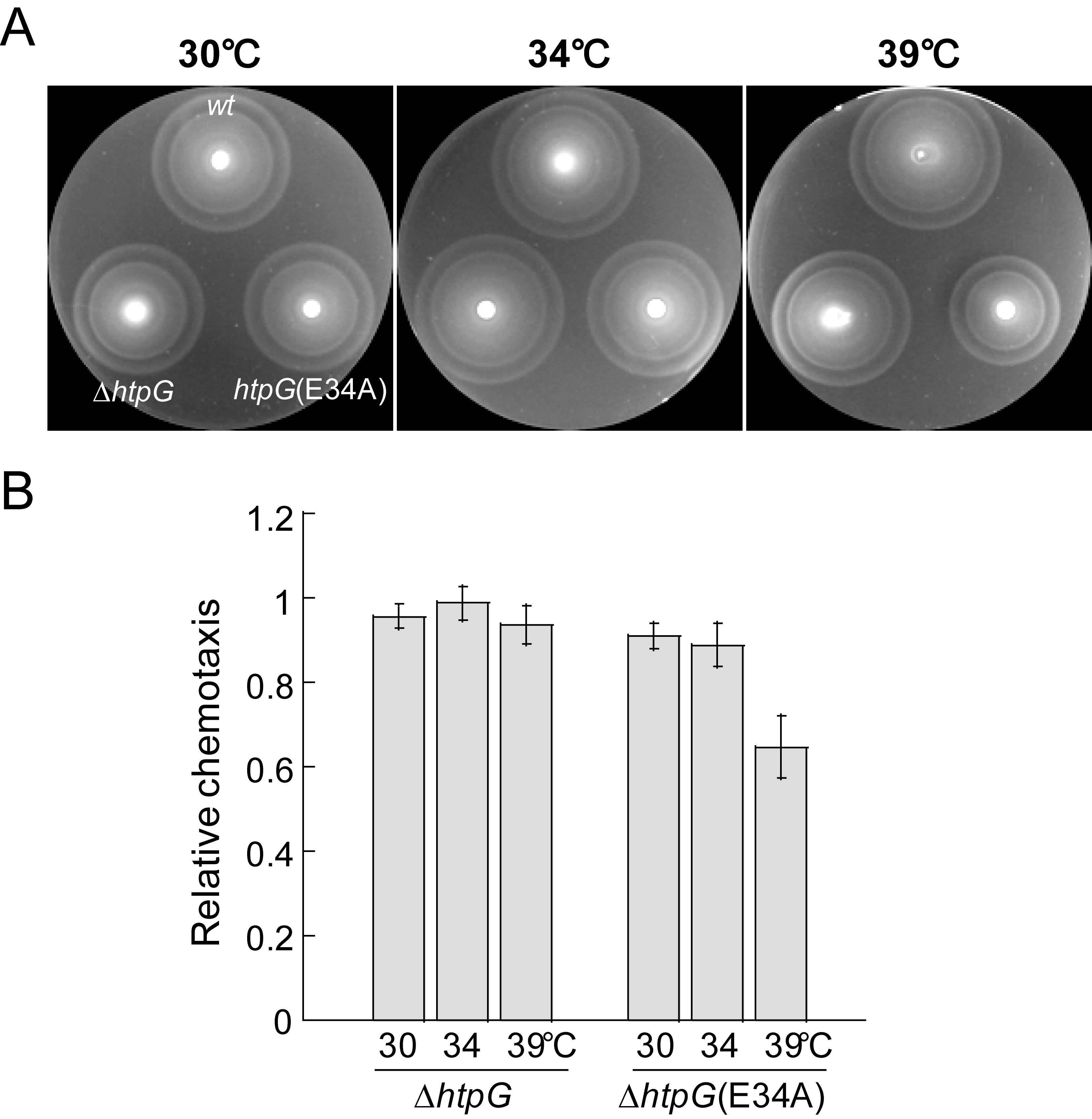

Supplement: Figure S5 — The htpG(E34A) mutant strain shows decreased motility/chemotaxis. (A) Plates were inoculated with the same amount of wild-type MG1655 (top), the ΔhtpG mutant (bottom left) and the htpG(E34A) mutant (bottom right) cells and incubated at indicated temperatures for 6 hr. (B) Relative motility of ΔhtpG and htpG(E34A) mutants, compared to wild type, at indicated temperatures, quantified by the diameter of the outer rings of spreading colonies. Error bars indicate standard errors from two replicates. (TIF) [file pgen.1003631.s006.tif]

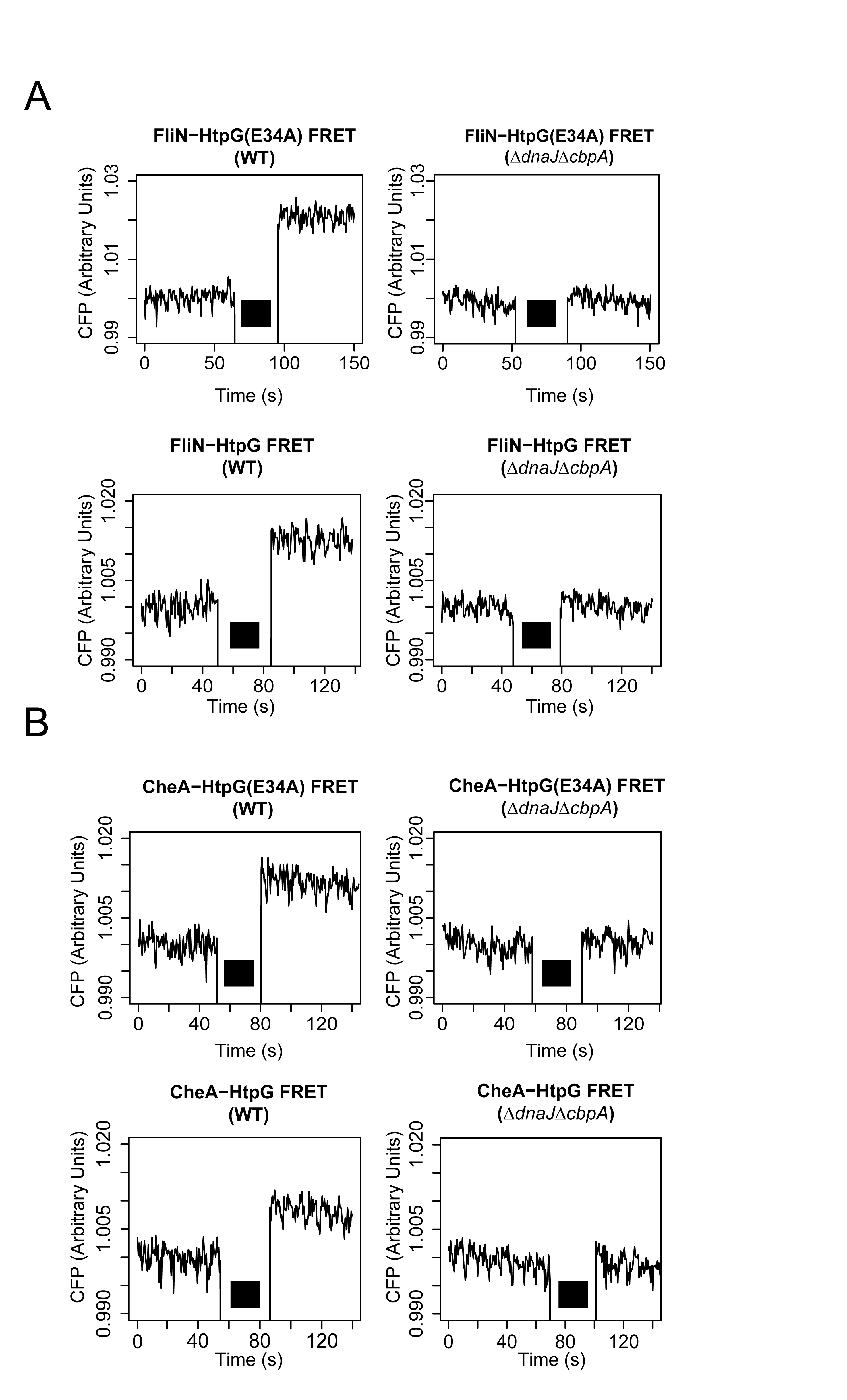

Supplement: Figure S6 — HtpG interactions with FliN and CheA are dependent on the DnaJ/CbpA/DnaK chaperone system. Acceptor photobleaching FRET was measured between HtpG and FliN (A) or CheA (B). In each panel, HtpG(E34A) (top row) and wild-type HtpG (bottom row) were assayed, and experiments were performed in both WT (left column) and ΔdnaJΔcbpA (right column) backgrounds. Y-axes are normalized in each case to the mean CFP signal before bleaching (first 45 s). Photobleaching begins at ∼50 s and lasts for 20 s (indicated by black bar). FRET interaction is indicated by a post-photobleaching increase in CFP signal above pre-photobleaching CFP signal (as observed in all experiments in the WT background). (TIF) [file pgen.1003631.s007.tif]
