# Supplementary material for: Genome-scale Co-evolutionary Inference Identifies Functions and Clients of Bacterial Hsp90
Source: PLoS Genet. 2013 Jul 11;9(7):e1003631. doi: 10.1371/journal.pgen.1003631 (PMC3708813; doi:10.1371/journal.pgen.1003631)
Supplement: Table S1 — Comparable results in Ciccarelli and Yarza trees across FDR thresholds. (DOC) [file pgen.1003631.s008.doc]

| **Table S1. Comparable results in Ciccarelli and Yarza trees across FDR thresholds.** | | | |
| --- | --- | --- | --- |
| **Yarza FDR threshold** | **FDR=0.1%** | **FDR=0.05%** | **FDR=0.01%** |
| **Genes passing threshold in Yarza tree** | 966 | 783 | 441 |
| ***Enriched function [KEGG Class] with p-values across FDR thresholds*** | | | |
| **Bacterial secretion system [PATHko03070]** | 3.20E-13* | 1.30E-14* | 3.42E-16* |
| **Secretion System [BRko02044]** | 2.44E-07* | 2.42E-08* | 1.03E-06* |
| **Bacterial motility proteins [BRko02035]** | 1.46E-06* | 3.38E-06* | 0.0096 |
| **Flagellar assembly [PATHko02040]** | 2.73E-10* | 1.17E-08* | 0.37 |
| ***Gene overlap with Ciccarelli tree*** | | | |
| **Genes in common** | 172 | 153 | 92 |
| **Hypergeometric p-value for overlap** | 6.67E-48* | 1.13E-48* | 1.38E-31* |
| **^*^significant at a 5% FDR among all hypergeometric tests for enrichment** | | | |
