# Supplementary material for: Genome-scale Co-evolutionary Inference Identifies Functions and Clients of Bacterial Hsp90
Source: PLoS Genet. 2013 Jul 11;9(7):e1003631. doi: 10.1371/journal.pgen.1003631 (PMC3708813; doi:10.1371/journal.pgen.1003631)
Supplement: Table S2 — E. coli strains and plasmids used in this study. (DOC) [file pgen.1003631.s009.doc]

| **Table S2. *E. coli* strains and plasmids used in this study.** | | | |
| --- | --- | --- | --- |
| **Strain** | **Relevant genotype** | **Background** | **Reference or source** |
| RP437 | wild type | - | Parkinson and Houts ^1^ |
| VS116 | *∆flhC* | RP437 | Sourjik and Berg ^2^ |
| MG1655 | wild type | - | Blattner et al. ^3^ |
| HL23 | *∆htpG* | MG1655 | This study |
| HL24 | *htpG::htpG(E34A)* | MG1655 | This study |
| MC4100 | wild type | - | Matthias Mayer, personal gift |
| HL25 | *∆dnaJ ∆cbpA* | MC4100 | Matthias Mayer, personal gift |
|  |  |  |  |
| **Plasmid** | **Relevant genotype** | **Background** | **Reference or source** |
| pHL13 | FliN-CFP expression | pDK79 | Li & Sourjik*,* 2011^4^ |
| pHL24 | HtpG-YFP expression | pTrc99a | Li & Sourjik*,* 2011^4^ |
| pHL35 | HtpG(E34A)-YFP expression | pTrc99a | This study |
| pHL52 | HtpG(E34A)-CFP expression | pDK79 | This study |
| pHL70 | HtpG-CFP expression | pDK79 | Li & Sourjik*,* 2011^4^ |
| pDK14 | CFP-CheW expression | pDK79 | Kentner *et al,* 2006^5^ |
| pDK19 | CheR-YFP expression | pTrc99a | Kentner & Sourjik*,* 2009^6^ |
| pDK29 | CheA-CFP expression | pDK79 | This study |
| pDK30 | CFP-CheA expression | pDK79 | This study |
| pDK36 | YFP-CheA^98-655^ (YFP-CheA_S_) expression | pTrc99a | Kentner & Sourjik*,* 2009^6^ |
| pDK49 | CheW-CFP expression | pDK79 | Kentner & Sourjik*,* 2009^6^ |
| pDK90 | YFP-CheA^509-655^ expression | pTrc99a | Kentner *et al,* 2006^5^ |
| pDK91 | YFP-CheA^326-655^ expression | pTrc99a | This study |
| pVS18 | CheY-YFP expression | pTrc99a | Sourjik and Berg, 2002^2^ |
| pVS64 | CheZ-YFP expression | pTrc99a | Liberman et al, 2004^7^ |
| pVS99 | CheB-YFP expression | pTrc99a | This study |
| pVS108 | CFP-CheA^156-655^ expression | pBAD33 | This study |
| pVS109 | CFP-CheA^259-655^ expression | pBAD33 | This study |
| pVS129 | CFP expression | pTrc99a | This study |
| pVS132 | YFP expression | pTrc99a | This study |
| 1: J Bacteriol, 1982. 151(1): p. 106-13 | | | |
| 2: Mol Microbiol, 2000. 37(4): p. 740-51 | | | |
| 3: Science, 1997. 277(5331): p. 1453-74 | | | |
| 4: Mol Microbiol, 2011. 80: p. 886–899 | | | |
| 5: Mol Microbiol, 2006. 61(2): p. 407-17 | | | |
| 6: Mol Syst Biol, 2009. 5: 238 | | | |
| 7: J Bacteriol, 2004. 186(19): p. 6643-6 | | | |
