# Supplementary material for: Genome-scale Co-evolutionary Inference Identifies Functions and Clients of Bacterial Hsp90
Source: PLoS Genet. 2013 Jul 11;9(7):e1003631. doi: 10.1371/journal.pgen.1003631 (PMC3708813; doi:10.1371/journal.pgen.1003631)
Supplement: Table S3 — Spreading of wild-type and ΔhtpG cells in soft-agar assays at 34°C and 42°C. (DOC) [file pgen.1003631.s010.doc]

| **Table S3. Spreading of wild-type and *ΔhtpG* cells in soft-agar assay at 34°C and 42°C^a^.** | | | |
| --- | --- | --- | --- |
| **Temperature** | **Strains** | **Center (%)** | **Outer edge (%)** |
| 34°C | MG1655 | 35±3 | 64±2 |
|  | MG1655*ΔhtpG* | 65±3 | 36±2 |
| 42°C | MG1655 | 41±3 | 63±2 |
|  | MG1655*ΔhtpG* | 59±3 | 37±2 |

^a^Assays were performed as described in Figure 4 and in Methods. Error bars indicate standard errors from four replicates.
