# Supplementary material for: Genome-scale Co-evolutionary Inference Identifies Functions and Clients of Bacterial Hsp90
Source: PLoS Genet. 2013 Jul 11;9(7):e1003631. doi: 10.1371/journal.pgen.1003631 (PMC3708813; doi:10.1371/journal.pgen.1003631)
Supplement: Table S4 — Acceptor photobleaching FRET interactions of chemotaxis components with HtpG(E34A). (DOC) [file pgen.1003631.s011.doc]

| **Table S4. Acceptor photobleaching FRET interactions of  chemotaxis components with HtpG(E34A).** | | |
| --- | --- | --- |
| **Chemotaxis component** | **HtpG(E34A) FRET in WT** | **HtpG(E34A) FRET in *∆flhC*** |
| **CheW-FP** | ++ | - |
| **FP-CheW** | ++ | + |
| **CheZ-FP** | ND | ++ |
| **CheY-FP** | ND | + |
| **CheR-FP** | ND | + |
| **CheB-FP** | ND | + |
| **CheA-FP** | ++ | ++ |
| **FP-CheA** | + | ++ |
| **FP-CheA^98-655^ (CheAs)** | ++ | ++ |
| **FP-CheA^156-655^** | ++ | ++ |
| **FP-CheA^259-655^** | ND | ++ |
| **FP-CheA^326-655^** | ND | - |
| **FP-CheA^509-655^** | ND | - |
| **++ Strong interaction; + Interaction; - No interaction; ND Not done.** | | |
