# Supplementary material for: Genome-scale Co-evolutionary Inference Identifies Functions and Clients of Bacterial Hsp90
Source: PLoS Genet. 2013 Jul 11;9(7):e1003631. doi: 10.1371/journal.pgen.1003631 (PMC3708813; doi:10.1371/journal.pgen.1003631)
Supplement: Table S5 — hsp90A presence and absence is associated with organismal traits in bacteria. (DOC) [file pgen.1003631.s012.doc]

| **Table S5. *hsp90A* presence and absence is associated with organismal traits in bacteria.** | | |
| --- | --- | --- |
| **Trait** | **p-value^1^** | **Species annotated^2^** |
| *Pathogenicity* | 0.036 | 140 |
| *Host-associated* | 0.046 | 146 |
| *Multiple habitats* | 0.0057 | 146 |
| *Terrestrial* | 0.045 | 146 |
| **1 :** average over 100 BayesTraits runsage over 100 BayesTraits runs | | |
| **2:** the number of species in the Ciccarelli tree with information for the trait in question. | | |
