# Supplementary material for: Genome-scale Co-evolutionary Inference Identifies Functions and Clients of Bacterial Hsp90
Source: PLoS Genet. 2013 Jul 11;9(7):e1003631. doi: 10.1371/journal.pgen.1003631 (PMC3708813; doi:10.1371/journal.pgen.1003631)
Supplement: Text S1 — Additional details on Hsp90 paralog distribution, consistency of BayesTraits runs, and robustness of co-evolutionary associations to choice of phylogeny. (DOC) [file pgen.1003631.s013.doc]

# Supporting Text

## Hsp90 paralog distribution

All bacterial Hsp90 paralogs were spread across multiple taxa, with gaps in their distribution (Figure S1C). *hsp90A* was widespread but particularly abundant in Proteobacteria, Clostridia, Actinobacteria, Chlorobi, and Chloroflexi. *hsp90B* was less common but dominant in Cyanobacteria and Bacteroidetes. *hsp90C* was relatively widespread but did not seem to be particularly enriched in any clade. While multiple Hsp90 paralogs could be observed in various species, *hsp90A* and *hsp90B* never co-occurred in the same species in our classification. This co-occurrence pattern and the distribution of *hsp90C* throughout the phylogeny suggest mostly vertical inheritance of *hsp90A* and *hsp90B* within clades and frequent horizontal transfers (and a potentially distinct functional role) of *hsp90C*. There are also a few instances of multiple copies of a single Hsp90 paralog within the same species (these are not displayed in Figure S1C).

## Consistency of BayesTraits runs

The stochastic nature of the BayesTraits maximum-likelihood algorithm allows for variation in rate parameter estimates from run to run. We accordingly confirmed that the results presented in the main text are robust to such variation. Specifically, we ran BayesTraits on the full set of 4645 genes 100 times, applying a 10% FDR threshold separately to each run, obtaining 100 sets of genes found to co-evolve with *hsp90A*. Examining these gene sets and their functional annotations, we found the results to be robust across runs. Specifically, the size of these sets ranged from 327 to 348 genes, with the vast majority of these genes (317) included in all sets. Functional enrichment was similarly consistent between the sets, with KEGG functional classes of flagellar assembly, bacterial motility proteins, and bacterial chemotaxis significantly enriched in all 100 runs. The bacterial secretion systems class was significantly enriched in 97 runs (and was just above the significance cutoff in the other 3). Considering this strong reproducibility between runs, in the main text we focused on the 327 genes that were found to co-evolve with *hsp90A* in at least 90% of runs. These genes are referred to throughout the text as ‘*hsp90A*-associated genes’.

We additionally evaluated the level of variation in estimated rate parameters. Overall, we found that rates are largely similar qualitatively between runs, with higher rates varying more from run to run than lower rates (see for example Figures 2C and 2D). Increasing the number of maximum-likelihood optimization attempts above the default did not appreciably affect this variability. Throughout the text we present the mean rates and standard deviations to communicate our estimated rates. To evaluate the accuracy of our ML-dependent approach, we also used BayesTraits’ Markov chain Monte Carlo (MCMC) mode with the reversible-jump option [50], which allows for parameter reduction, to estimate our gain and loss rate parameters for a small subset of genes (see Methods). We found that ML-based predictions of rates are highly consistent with MCMC estimates (Spearman’s rho = 0.91; p < 2.2 * 10^-16^).

We similarly examined the effect of BayesTraits variation on our ability to consistently classify genes into specific co-evolutionary models. We again ran BayesTraits 100 times, generating each of the four distinct evolutionary models for all 327 *hsp90A*-associated genes (see Methods). We independently applied AIC for each gene and for each of these 100 replicates to determine which model fit each gene best in each run. Genes for which at least 90 of the 100 runs agreed on one of these four models were classified with this model. This scheme was able to classify 312 of the 327 *hsp90A*-associated genes into either the mutual dependence model (model 2; Methods) or one of the unidirectional dependence models (models 3 and 4). Specifically, all bacterial secretion genes and all flagellar genes were successfully classified (see main text and Table 1 for a detailed discussion of these genes).

We used a similar approach to estimate the impact of run-to-run variation on the study of *hsp90A* co-evolution with organismal traits. The traits of host-association, multiple habitat preference, and pathogenicity were all found to co-evolve with *hsp90A* in all 100 runs, and the trait of terrestriality was found to co-evolve with *hsp90A* 99 times of 100. *hsp90A* was always found to be dependent on multiple habitat preference and pathogenicity, whereas both terrestriality and host-association had a mutually dependent relationship with *hsp90A*.

Finally, since our client prediction method is based on estimated rates (see Methods), we evaluated the sensitivity of our method to variation in rate estimation. To this end, we recalculated putative client index (PCI) values independently for each of the 100 runs above. For each run we recorded the 20 genes with the lowest PCI values and examined the variation observed from run to run in this set of potential clients. Overall, only 26 unique genes were identified as potential clients by any run, with 12 genes identified as clients in all 100 runs and 18 genes identified as clients in at least 90 of the runs, indicating high reproducibility. In the main text, we define these 18 genes found in at least 90 runs as putative clients and report their mean PCI values (see Table 2). Figure 3 illustrates the distribution of mean PCI values across all 327 *hsp90A*-associated genes.

## Robustness of co-evolutionary associations to choice of phylogeny

One potential caveat of our analysis is its strong dependency upon a specific phylogeny. Accordingly, while the Ciccarelli tree used throughout our analysis is well-established, commonly used, and believed to be robust, we examined whether our results hold with a different phylogenetic tree. To this end, we repeated the analysis described in the main text using a significantly larger tree (including 797 species) that was constructed by a fundamentally different method [30] (termed here ‘*Yarza tree*’). We find that the p-values obtained using this larger tree are correlated with those reported in the main text using the Ciccarelli tree (p < 2.2E-16; Spearman correlation test), but are generally much smaller (requiring the use of a smaller FDR threshold). While the set of *hsp90A*-associated genes found in the Yarza tree is generally larger than the set of genes found in the Ciccarelli tree, there is a significant overlap between the sets (Table S1). Moreover, examining functional enrichment in the Yarza-derived *hsp90A*-associated gene set, we find the same set of functional categories as those found in the Ciccarelli tree across a variety of FDR thresholds (Tables S1).

**Effect of alternate *hsp90* paralogs on co-evolution**

There are 20 species in the Ciccarelli tree that contain either *hsp90B, hsp90C,* or both. In the analysis presented in the main text we focused on *hsp90A*, and therefore treated these species similarly to those species that lack *hsp90* altogether. To confirm that the inclusion of these species did not impact our analysis of genes that co-evolve with *hsp90A*, we repeated our analysis after pruning the Ciccarelli tree to remove these species. The pruned tree included a total of 128 species, with 4399 genes that passed our filters. Clearly, excluding these 20 species reduced the statistical power of our analysis (a phenomenon that we also observed in using a larger tree, see above). We accordingly increased the FDR threshold used in our pipeline to 25% to obtain a co-evolving gene set of similar size (301 genes vs. 327 genes with the full set). Functional enrichments in this gene set were similar to enrichments obtained using the full tree (Flagellar assembly: 19/39 genes, bacterial motility proteins: 27/108 genes, and bacterial secretion: 16/64 genes; compare to Table 1), with very similar levels of enrichment. The enrichment of chemotaxis genes was very similar to that observed in the full set (7/26 genes, p = 0.00037), but did not reach significance with a 5% FDR (for detecting enrichment among all possible functions), potentially due to the smaller sample size. We conclude that inclusion or exclusion of species containing the alternate *hsp90* paralogs did not qualitatively bias our analysis, though reduction of phylogenetic coverage reduces our power to detect associations in a genome-wide fashion.
